# Is the Urban-Rural Divide Affectively Polarised? Comparative Evidence from Nine European Countries

**DOI:** 10.1177/00104140251369317

**Published:** 2025-08-29

**Authors:** Sven Hegewald, Dominik Schraff

**Affiliations:** 1 27219 ETH Zurich, Zurich, Switzerland; 2 1004 Aalborg University, Aalborg, Denmark

**Keywords:** urban-rural divide, affective polarisation, transnational cleavage, place-based resentment, place-based identity

## Abstract

Recent studies in the United States and Europe have documented a growing divergence in voting behaviour and political attitudes between cities and the countryside. However, we still lack systematic evidence on the extent to which this urban-rural divide is also affectively polarised. To shed light on this, we advance the concept of place-based affective polarisation, which we define as the difference between in-group and out-group affect in relation to place-based groups. Drawing on original survey data from nine European countries, we show that place-based affective polarisation is substantial along the urban-rural divide and associated with strong feelings of place-based resentment and identity. Furthermore, we find that higher levels of place-based affective polarisation correlate with support for GAL parties (green, alternative, libertarian) among urbanites and support for TAN parties (traditional, authoritarian, nationalist) among ruralites. Overall, our findings point to a strong political cleavage between urban and rural areas in several European countries.

## Introduction

The urban-rural divide has woken up from its dormancy. In the United States, the countryside has become a stronghold of the Republican party, while people living in cities are voting overwhelmingly for the Democrats (e.g., Gimpel et al., 2020; Rodden, 2019; Scala & Johnson, 2017; Taylor et al., 2024). Similarly, in Europe, support for radical right and new left parties increasingly clusters in rural and urban areas (Huijsmans & Rodden, 2025). Comparable patterns can be found in connection to different political attitudes and support for the political system. In general, ruralites tend to hold more nationalistic attitudes than urbanites, especially concerning immigration and European integration (e.g., Huijsmans et al., 2021; Jennings & Stoker, 2016; Maxwell, 2019, 2020). Besides this, rural residents also harbour lower levels of trust in political institutions and are less satisfied with how democracy works (e.g., Hegewald, 2024b; Lago, 2022; McKay et al., 2021; Mitsch et al., 2021; Stein et al., 2021; Zumbrunn, 2024a).

While these empirical patterns are well documented by now, their explanations remain considerably debated in the literature. One branch of studies typically focuses on sorting dynamics, either arguing that residential choice is politically motivated (e.g., Bishop, 2008; Gimpel & Hui, 2015) or that socio-demographic factors confound the correlation between political preferences and location (e.g., Gallego et al., 2016; Martin & Webster, 2020; Maxwell, 2019). Other studies, by contrast, emphasise place-based grievances, suggesting that territorial political divisions are rooted in a backlash against political elites in places left behind by globalisation (e.g., Broz et al., 2021; Colantone & Stanig, 2018a, 2018b; Dijkstra et al., 2020; Iversen & Soskice, 2019; Rodríguez-Pose, 2018; Schraff & Pontusson, 2024). Besides this, several other studies also revert to people’s place-based identities to explain regional differences in political behaviour (e.g., Bolet, 2021; Bornschier et al., 2021; Fitzgerald, 2018; Zollinger, 2024b). Works on place-based resentment bring these last two approaches together, arguing that it is the intersection of place-based grievances and identities that explains people’s political choices and attitudes along the urban-rural divide (e.g., Cramer, 2016; Huijsmans, 2023a, 2023b; Jacobs & Munis, 2023; Lunz Trujillo & Crowley, 2022; Munis, 2022).

However, despite this wealth of existing explanations, there is little systematic evidence on the extent to which the urban-rural divide is also *affectively* polarised (but see Lyons & Utych, 2023; Zumbrunn, 2025). Nonetheless, addressing this question is crucial for two primary reasons. First, cleavages are typically defined as incorporating a structural element that connects to party politics through a “set of values and beliefs which provides a sense of identity” (Bartolini & Mair, 1990, p. 215). Linking structural to political divides, identities anchor individuals both socially and politically (Bornschier, 2009; Westheuser & Zollinger, 2025). A strong affective basis is, therefore, a necessary condition for the development of a full-fledged cleavage (e.g., Borbáth et al., 2023). Consequently, investigating the extent to which the urban-rural divide is affectively polarised is essential to detect whether differences between cities and the countryside represent a relevant line of political conflict. Second, as demonstrated by the literature on affective partisan polarisation, partisanship as a social identity fuels political conflict when a strong attachment to co-partisans coincides with an aversion directed at partisans from the other side of the aisle (Iyengar et al., 2012, for an overview, see Iyengar et al., 2019). In this regard, examining affective polarisation between urbanites and rural residents is not only critical for cleavage detection but also promises to considerably deepen our understanding of how place gives rise to political divisions.

We, therefore, advance the concept of place-based affective polarisation. Inspired by the literature on affective partisan polarisation, we define place-based affective polarisation as a pronounced bias of individuals to like people from their own place more than others living in different places. We thus conceive place-based affective polarisation as the difference in affect towards place-based in-groups and out-groups. Applying this concept to the urban-rural divide, we focus on the tendency of urbanites (ruralites) to like members from their urban (rural) in-group more than members of their rural (urban) out-group. Building on insights from social psychology, in particular, social identity theory (Tajfel & Turner, 1979), realistic group conflict (Sherif et al., 1961), and integrated threat theory (Stephan & Stephan, 2000), we propose that feelings of place-based resentment and identity should correlate with place-based affective polarisation. Furthermore, taking a group-based approach to partisanship (e.g., Achen & Bartels, 2016; Green et al., 2004; Huddy et al., 2015), we argue that place-based affective polarisation should be associated with voting for GAL (green, alternative, libertarian) and TAN parties (traditional, authoritarian, nationalist), which occupy opposing positions on the transnational cleavage in European politics (e.g., Dassonneville et al., 2024; Hooghe et al., 2002, 2025; Hooghe & Marks, 2018; for an overview, see Marks et al., 2021).

We test our arguments by drawing on original survey data from nine European countries (Czech Republic, Denmark, France, Germany, Greece, Hungary, Italy, Poland, and Spain). We find that place-based affective polarisation is substantial along the urban-rural divide. Analogue to affective partisan polarisation, many individuals in our data tend to like their place-based in-groups over their place-based out-groups. Although this phenomenon is more pronounced for ruralites than urbanites, both groups exhibit place-based affective polarisation in all countries under investigation. Moreover, as expected, place-based resentment and identity positively correlate with place-based affective polarisation. Again, these associations are generally stronger for ruralites than urbanites. Finally, we document that urbanites who like their own kind over ruralites, exhibit higher levels of support for GAL parties. Conversely, among ruralites, increasing levels of place-based affective polarisation tend to bolster support for TAN parties.

Our study makes five central contributions. First, we provide evidence that the urban-rural divide constitutes a significant line of political conflict in Europe. Specifically, our findings reveal a substantive affective component underpinning political divisions between cities and the countryside in several European countries. Given the importance of this affective dimension for the development of a full-fledged cleavage, our study offers critical evidence that the urban-rural divide serves as a relevant fault line in European politics, particularly when it comes to conflicts over transnationalism.

Second, we extend the literature on place-based identity, which predominantly concentrates on people’s attachments to place-based in-groups. However, only when looking at sentiments directed at place-based in-groups and out-groups in conjunction, we get a complete picture of how place-based antagonisms fuel political divisions. We are able to show this empirically. Even when controlling for in-group affect (out-group affect), out-group affect (in-group affect) remains a relevant explanatory variable for voting behaviour. Furthermore, the importance of in-group affect outweighs that of out-group affect among urban residents, while the opposite is true for rural residents. We would have missed these nuances if we had focused on in-group attachments only.

Third, we offer an alternative approach to measuring polarisation along the urban-rural divide. In our view, a key limitation of existing measurements of place-based resentment is the hard-wiring of a limited set of grievances into these measures (e.g., Claassen et al., 2025; Hegewald, 2024b; Huijsmans, 2023a, 2023b; Munis, 2022). These typically address conflicts over economic resources, political representation, and culture. While this approach certainly adds theoretical richness, it also introduces strong assumptions about which structural conflicts are most relevant to political divisions along the urban-rural divide. This limitation is particularly problematic in the European context, given that many of these measures are rooted in ethnographic work conducted in the United States (e.g., Cramer, 2016). As a result, existing measures risk overlooking other significant structural conflicts. For instance, while economic grievances in these measures are often framed around taxation, other economic issues, such as housing, also have a geographic dimension that has the potential to fuel conflict between urbanites and ruralites (e.g., Adler & Ansell, 2020; Ansell, 2014, 2019).

By contrast, our measure of place-based affective polarisation adopts a different approach by remaining agnostic to the specific structural conflicts underlying the urban-rural divide. Instead, it directly captures the degree of affective polarisation between urbanites and ruralites without reverting to a predetermined set of grievances. We believe this offers a more flexible and adaptable measurement strategy. Unlike existing measures of place-based resentment, it avoids priming survey participants with specific structural conflicts. In this sense, our measure could be considered a more conservative approach to capturing polarisation along the urban-rural divide. Moreover, this should also make our measurement more adaptable in comparative research settings, where the relevance of different structural conflicts may vary significantly across country contexts.

Fourth, we provide some much-needed comparative evidence to a literature that tends to be dominated by single-country studies. Many works focus either on the United States (e.g., Cramer, 2016; Jacobs & Munis, 2023; Lunz Trujillo & Crowley, 2022; Lyons & Utych, 2023; Munis, 2022), Switzerland (e.g., Bornschier et al., 2021; Zollinger, 2024b; Zumbrunn, 2024a, 2024b, 2025), or the Netherlands (e.g., Huijsmans, 2023a, 2023b). Conversely, we show that many of our hypotheses hold across a diverse range of contexts. However, we can only speculate why we find some diverging patterns, especially in Southern European countries such as Greece and Spain. Nevertheless, our study lays out a comparative research agenda that should investigate these differences more thoroughly.

Fifth, we contribute to an emerging literature showing that affective polarisation can transcend partisan identities. Similar to studies extending the concept to opinion- (Hobolt et al., 2021) and education-based groupings (Van Noord et al., 2025), we show that place-based groups can also give rise to affective polarisation. In this regard, we relate to recent studies in the United States (Lyons & Utych, 2023) and Switzerland (Zumbrunn, 2025), already providing first evidence of affective polarisation along the urban-rural divide.

The remainder of this study proceeds as follows. After reviewing the literature on the return of the urban-rural divide, we take stock of the existing explanations of territorial political divisions. We then present our theoretical arguments, data, and results. We conclude by summarising our main findings and highlighting some areas for future research.

## The Return of the Urban-Rural Divide

In Lipset and Rokkan’s (1967) original conceptualisation, the urban-rural cleavage was related to a conflict between a landed elite and an urban entrepreneurial class that came to rise during the Industrial Revolution. Although its roots can be traced back to medieval times, in most European countries, divisions between urbanites and ruralites have not resulted in a persistent line of political conflict (Gallagher et al., 2020). Consequently, following its heyday at the end of the 19^th^ and beginning of the 20^th^ century, the urban-rural divide became largely dormant, which meant that it only played a minor role in the analysis of political attitudes and behaviour.

However, more recently, a rapidly growing body of literature has reinstated an interest in studying political divisions between cities and the countryside (for an early overview, see Ford & Jennings, 2020). In the United States, various studies show that rural areas tend to vote overwhelmingly Republican, while cities have largely become strongholds of the Democratic party (e.g., Gimpel et al., 2020; Rodden, 2019; Scala & Johnson, 2017; Taylor et al., 2024). In Europe, comparative evidence also indicates a re-emergence of the urban-rural divide, which seems to be mostly driven by increasing support for radical right and new left parties in rural and urban areas, respectively (Huijsmans & Rodden, 2025).

A similar tendency can be detected in citizens’ political attitudes. In England, for example, Jennings and Stoker (2016) show a growing divergence between urban centres with a more global, pluralist outlook and more provincial areas becoming increasingly pessimistic about issues concerning European integration and multiculturalism. Likewise, focusing specifically on immigration attitudes, Maxwell (2019, 2020) documents that individuals from major European cities have much more favourable views about immigration than rural residents. Similarly, Huijsmans et al. (2021) find a growing divide in various cultural attitudes between urban and rural areas in the Netherlands, owing to a dynamic where public opinion of people living in urban places has become more cosmopolitan than the rest of the country.

Besides this, an urban-rural divide is also visible in various measures of political support. Studies commonly find that political trust is higher among urban residents compared to rural residents, with this divergence having intensified over time in several European countries (e.g., Hegewald, 2024b; Kenny & Luca, 2021; McKay et al., 2021; Mitsch et al., 2021; Stein et al., 2021; Zumbrunn, 2024a). Beyond political trust, recent research also highlights lower levels of satisfaction with democracy and political efficacy in rural places, suggesting that rural residents might feel especially alienated from democratic processes (e.g., Del Horno et al., 2024; Lago, 2022; Rowland et al., 2024). This general sentiment is further underlined by Zumbrunn and Freitag (2023), who show that individuals living in rural areas tend to be more supportive of authoritarian forms of government.

In sum, all of these studies document a puzzling awakening of the urban-rural divide. Cities and the countryside seem to gradually drift apart in their voting behaviour, political attitudes, and support for the political system more generally. While these empirical trends are becoming increasingly clear by the day, what explains these divisions remains a matter of considerable debate.

## Existing Explanations of Territorial Political Divisions

Within the broader literature on geographic divides, at least four main arguments explaining territorial political divisions can be distinguished. The first argument focuses on sorting dynamics. In this branch of the literature, some works argue that people generally prefer to live in places that are predominately inhabited by others with similar political attitudes (e.g., Bishop, 2008; Gimpel & Hui, 2015; Ihlanfeldt & Yang, 2024a; McCartney et al., 2024; Motyl et al., 2014). For example, McCartney et al. (2024) show that partisans are more likely to sell their homes when supporters of a different party move in next door. Furthermore, Gimpel and Hui (2015) find that receiving information about properties being located in a co-partisan neighbourhood tends to increase positive evaluations of these properties. However, even though people appear to be more satisfied when living together with co-partisans, other factors such as neighbourhood safety or school quality are usually found to trump partisanship as an explanation for residential choice (Hui, 2013). Moreover, people’s ability to move is often constrained by the supply of affordable housing in co-partisan areas (Mummolo & Nall, 2017).

In light of this, other studies propose that neighbourhood characteristics and socio-demographic factors confound the correlation between political preferences and location (e.g., Gallego et al., 2016; Hoogerbrugge & Burger, 2022; Ihlanfeldt & Yang, 2024b; Martin & Webster, 2020; Maxwell, 2019). Instead of consciously choosing a place to live according to their political outlooks, Martin and Webster (2020) show that partisans tend to sort into places based on non-political considerations that correlate with partisanship. This dynamic is further illustrated by Maxwell (2019), who finds that the urban-rural divide in immigration attitudes is primarily driven by the self-selection of highly educated, pro-immigration professionals into urban areas. This suggests that residential choice is endogenous to people’s political behaviour as relocation decisions are based on considerations associated with political orientations. In this sense, similar to works that view relocation as politically motivated, these studies also emphasise the importance of geographic sorting dynamics as an explanation for territorial political divisions.

The second argument typically revolves around place-based grievances, usually rooted in economic considerations. Studies in this strand of the literature often propose that globalisation has triggered a backlash against political elites in regions suffering from the adverse effects of a globalised economy (e.g., Broz et al., 2021; Colantone & Stanig, 2018a, 2018b; Cremaschi et al., 2024; Dijkstra et al., 2020; Iversen & Soskice, 2019; Rodríguez-Pose, 2018; Rodríguez-Pose et al., 2023; Rodríguez-Pose & Ketterer, 2020; Schraff & Pontusson, 2024). Perhaps most prominently, Rodríguez-Pose (2018) outlines how persistent economic decline in many areas around the world has led voters living in these places to support radical right political candidates such as Donald Trump in the United States or Marine Le Pen in France. Similarly, Broz et al. (2021) show that a significant increase in regional inequalities underpins the emergence of radical right strongholds in left-behind places. Furthermore, Colantone and Stanig (2018a) demonstrate that the Brexit vote in the United Kingdom was strongly driven by citizens’ sociotropic concerns about the economic state of their area, with those living in regions hit hardest by globalisation exhibiting a higher propensity to have voted to leave the European Union. In connection to this, several recent studies have also shown that declining quality of governance and public service deprivation further fuel support for the radical right in places left behind (e.g., Cremaschi et al., 2024; Rodríguez-Pose & Ketterer, 2020). Overall, what this second branch of studies has in common is a focus on individuals from areas struggling to keep up with an increasingly globalised economy, leading them to cast their ballots for radical right political parties and candidates.

The third argument presented in the literature then draws on insights from social and environmental psychology, conceiving people’s place of living as the basis for a social identity (e.g., Proshansky, 1978; Proshansky et al., 1983; Tajfel, 1974; Tajfel & Turner, 1979). According to Tajfel’s (1981) influential definition, a “social identity” relates to “that *part* of an individual’s self-concept which derives from his knowledge of his membership of a social group (or groups) together with the value and emotional significance attached to that membership” (p. 255, emphasis in original). While individuals can belong to and identify with various social groups, studies in this area of the literature tend to emphasise the place of residence as a significant marker of group membership, giving rise to pronounced place-based identities that structure political behaviour. For instance, Fitzgerald (2018) proposes that voting for the radical right is strongly related to citizens’ emotional ties to their local community. In particular, individuals with a strong attachment to their place of residence are found to be likely supporters of radical right parties, viewing them as potential defenders of their home locality. Related to this, Bolet (2021) suggests that local pub closures foster the degradation of place-based identity, which strongly correlates with support for the radical right in the United Kingdom. Moreover, studying voting behaviour in Switzerland, Bornschier et al. (2021) and Zollinger (2024b) underscore the centrality of place-based identities in explaining political divisions along the urban-rural divide. They demonstrate that individuals with strong attachments to urban places are more likely to vote for new left parties, whereas those who feel a strong sense of belonging to rural areas tend to be more supportive of the radical right. In this sense, to explain territorial political divisions, studies in this third stream of the literature highlight people’s emotional connections to their place of living rather than place-based economic grievances or geographic sorting.

Lastly, the fourth explanation in the literature relates to a feeling of place-based resentment, which can be viewed as an approach that brings together place-based grievances and place-based identity. According to Cramer’s (2016) groundbreaking ethnographic work conducted in rural Wisconsin, place-based resentment describes a feeling where “an identity rooted in place … is infused with a sense of distributive injustice” (p. 12). In this regard, place-based resentment is located at the intersection of place-based identities and place-based grievances, both of which come causally prior to place-based resentment (Munis, 2022). Commonly, place-based resentment is conceptualised in relation to three different sources of place-based grievances: (a) the perception that one’s place is getting fewer resources than it deserves, (b) the belief that policymakers do not pay enough attention to the interests of residents from one’s place, and (c) the feeling that the unique lifestyles of people living in one’s place are disrespected by people from other places (Claassen et al., 2025; Cramer, 2016; Huijsmans, 2023a, 2023b; Munis, 2022). In particular, studies in the United States document pronounced political effects of place-based resentment. Lunz Trujillo and Crowley (2022), for example, show that rural residents who regard their place as underrepresented and disrespected are more prone to support Donald Trump. Likewise, Jacobs and Munis (2023) find that place-based resentment strongly predicted voting for the Republican party in recent elections. Beyond the context of the United States, Huijsmans (2023a) provides evidence that place-based resentment mediates the relationship between people’s place of living and their attitudes concerning populism and immigration in the Netherlands. In this sense, to understand place-based divisions in politics, the place-based resentment approach draws attention to an interplay between attachment to place and a feeling that one’s place is treated unfairly.

## Conceptualising Place-Based Affective Polarisation

Despite the richness of the existing explanations reviewed above, we nevertheless have limited systematic evidence regarding the degree of affective polarisation along the urban-rural divide (but see Lyons & Utych, 2023; Zumbrunn, 2025). Such evidence, however, is critical to detect whether divisions between cities and the countryside represent a significant line of political conflict. Arguably, following the established definition of a cleavage comprising a structural, affective, and organisational component, a pronounced degree of affective polarisation along the urban-rural divide can be regarded as a necessary condition for the existence of a fully developed cleavage (e.g., Bartolini & Mair, 1990; Borbáth et al., 2023). This affective component acts as a bridge between the structural and organisational elements, reflecting the “the self-awareness of the social group(s) involved” (Bartolini, 2000, p. 17). In this regard, affect plays a critical role by embedding individuals in social structure on the one hand and party politics on the other hand (Bornschier, 2009; Westheuser & Zollinger, 2025).

We, therefore, set out to investigate the extent to which the urban-divide is affectively polarised, advancing the concept of place-based affective polarisation. Inspired by the literature on affective partisan polarisation (e.g., Iyengar et al., 2012, 2019), we formally define place-based affective polarisation as *an individual’s propensity to like people from their own place more than people from a respective geographic out-group*. We thus conceive place-based affective polarisation as denoting the difference between in-group and out-group affect in relation to place-based groups. We are keeping this definition intentionally broad so it can be applied to all kinds of different territorial divides. However, in this study, we focus on the urban-rural divide as a geographic fault line that has gained increasing prominence in recent years, as evidenced by the numerous studies reviewed in the previous sections.^1^ Therefore, we concentrate on the tendency of urbanites (ruralites) to like members from their urban (rural) in-group more than members of their rural (urban) out-group.

Similar to affective partisan polarisation, place-based affective polarisation draws heavily on classic works in social psychology. According to social identity theory, the development of a social identity boils down to three interconnected processes (e.g., Tajfel, 1974; Tajfel, 1981; Tajfel & Turner, 1979; for an overview, see Brewer, 2019). The first is social categorisation, where individuals classify themselves into in-groups and others into out-groups. The second is social identification, which relates to the incorporation of group membership into one’s concept of self. The third and final process is social comparison, involving a positive evaluation of one’s in-group against a given out-group. As such, a major function of a social identity is to provide individuals with a sense of self-esteem (Tajfel & Turner, 1979).

The place-based identity approach discussed in the previous section focuses on the first and second processes involved in the development of social identities (Zumbrunn, 2024b). While these works demonstrate the important consequences of place-based identities for political attitudes and behaviour, they generally pay less attention to the aspect of social comparisons by predominantly concentrating on attachments to people’s place-based in-groups. Crucially, this limits our understanding of how place-based identities can become politically divisive. As forcefully illustrated by the literature on affective partisan polarisation, partisanship as a social identity underpins political conflict when strong emotional bonds towards co-partisans coincide with a deep-seated aversion directed at partisans from the other side of the aisle (e.g., Iyengar et al., 2012, 2019). Over time, this can lead to a situation in which citizens are increasingly divided along partisan lines, substantially undermining the functioning of democratic political systems (e.g., Hetherington & Rudolph, 2015; Kingzette et al., 2021). In light of these insights, it thus appears central to consider both sentiments directed at place-based in-groups and place-based out-groups if we want to explain why place gives rise to political divides.

One approach to integrating sentiments directed at place-based in-groups and out-groups can be found in the literature on place-based resentment. On the one hand, as discussed above, place-based resentment encompasses a strong sense of place-based identity, while, on the other hand, it also involves place-based grievances, which essentially originate from a comparison between place-based in-groups and out-groups (Zumbrunn, 2024b). Although we regard this combination of the different processes involved in the development of social identities as a strength of place-based resentment, we also think that it presents significant challenges. Situating place-based resentment at the intersection of place-based identities and place-based grievances effectively hard-wires a rather narrow set of structural conflicts over economic resources, political representation, and culture into the concept and, by extension, its accompanying empirical measures (e.g., Claassen et al., 2025; Hegewald, 2024b; Huijsmans, 2023a, 2023b; Munis, 2022). While this certainly adds theoretical depth, it also introduces strong assumptions about which structural conflicts matter most for political divisions between urbanites and ruralites. This limitation warrants particular caution when applying this concept to the European context, given that many current place-based resentment measures are grounded in ethnographic fieldwork conducted in the United States (e.g., Cramer, 2016). More specifically, an uncritical application of place-based resentment to contexts outside of the United States risks overlooking other key conflicts, such as housing issues, which also have a geographic dimension and can drive urban-rural tensions (e.g., Adler & Ansell, 2020; Ansell, 2014, 2019).

Place-based affective polarisation, by contrast, remains agnostic to the specific structural conflicts underpinning the urban-rural divide while, at the same time, integrating sentiments directed at place-based in-groups and out-groups. This allows the concept to travel smoothly across different country contexts, which is particularly important in comparative research settings. Empirically speaking, our approach also has the advantage of preventing respondents from thinking about the urban-rural divide in terms of a given set of structural conflicts that were determined a priori. In this sense, our measure could be considered a more conservative approach to capturing polarisation along the urban-rural divide.

## Hypotheses

Departing from this conceptualisation of place-based affective polarisation, we now derive a number of theoretical expectations. Our first expectation is that place-based affective polarisation is asymmetric between urban and rural residents. We argue that rural residents should exhibit a particular tendency to distinguish their rural in-group positively by attributing negative characteristics to urbanites. Although this is only one way to derive self-esteem from a social identity, it is an especially attractive option for members of lower-status groups such as rural residents (Huddy, 2003). Regarding subjective social status, Vigna (2023), for instance, finds that ruralites often view themselves as occupying the bottom of the social ladder, while urban residents tend to gravitate towards the top. These differences should make it more likely for ruralites to harbour stronger feelings of place-based affective polarisation. Another reason for this asymmetry might also relate to geographic sorting dynamics similar to those reviewed above. Europeans are increasingly moving into cities for work, studying, and other reasons, thereby actively choosing to live in urban over rural places (e.g., Florida, 2002, 2003; Glaeser, 2011). This selective migration might lead a growing number of urbanites who used to live in rural areas to be divided in terms of their loyalties. For instance, individuals who were socialised in rural areas but now live in urban places should be less likely to exhibit strong place-based affective polarisation.

**H**_**1**_: Place-based affective polarisation is more pronounced among rural residents than urban residents.

Place-based resentment, we contend, should correlate with place-based affective polarisation (Zumbrunn, 2025). Perceptions of threat and conflict over scarce resources have been linked to out-group negativity as well as in-group bias (for overviews, see Riek et al., 2006; Brewer, 2019). Early works in the tradition of realistic group conflict theory (Campbell, 1965) have documented pronounced in-group favouritism and out-group hostility in situations where groups have to compete (Sherif et al., 1961; Sherif & Sherif, 1969). Expanding this theoretical approach, Stephan and Stephan’s (2000) integrated threat theory posits that out-group derogation is particularly strong when an in-group perceives a threat from an out-group to its position of power, material well-being, or the norms and values for which it stands.^2^ With this in mind, we conceive the triad of place-based grievances that are central to place-based resentment as perceptions of threat. Essentially, we propose that place-based resentment encompasses the perception of a place-based in-group being threatened by a place-based out-group with regard to its resources, representation in politics, and respect (Cramer, 2016). Following from this, strong feelings of place-based resentment should increase place-based affective polarisation. However, since urbanites tend to harbour lower levels of place-based resentment than ruralites (e.g., Borwein & Lucas, 2023; Munis, 2022; Zumbrunn, 2024b), we expect that the correlation between place-based resentment and affective polarisation is more pronounced among individuals living in rural areas.

**H**_**2a**_: The stronger individuals’ place-based resentment, the higher their level of place-based affective polarisation.
**H**_**2b**_: The correlation between place-based resentment and place-based affective polarisation is more pronounced among rural residents than urban residents.

Besides place-based resentment, place-based affective polarisation might also correlate with individuals’ place-based identities (Zumbrunn, 2025). Following the minimal group paradigm, simply being a member of a place-based in-group might already be sufficient to induce place-based affective polarisation. Pioneering experiments in social psychology have shown that even when individuals are assigned to explicitly arbitrary social groups, they still tend to favour their in-group over their out-group when asked to allocate resources between the two (e.g., Billig & Tajfel, 1973; Tajfel, 1970; Tajfel et al., 1971; for an overview, see Diehl, 1990). Following from this, urbanites and ruralites might already be biased towards their respective in-group by their mere group membership alone. However, this dynamic is not inevitable and generally heightened under certain conditions (Brewer, 1999). Most importantly, the effect of group membership tends to be mediated by the degree of identification with the in-group (Grieve & Hogg, 1999). In particular, research in political psychology highlights that in-group bias, as well as negative sentiment towards an out-group, crucially depend on the strength of in-group attachments (for an overview, see Huddy, 2001). Therefore, the stronger urbanites (ruralites) feel attached to their urban (rural) in-group, the more they might favour this group over a rural (urban) out-group. Nevertheless, this tendency should again be more pronounced for ruralites as they tend to exhibit stronger place-based identities than urbanites (e.g., Haffert et al., 2024).

**H**_**3a**_: The stronger individuals’ place-based identity, the higher their level of place-based affective polarisation.
**H**_**3b**_: The correlation between place-based identity and place-based affective polarisation is more pronounced among rural residents than urban residents.

Tentative evidence from the United States and Switzerland already lends credence to some of our arguments. Among American voters, Lyons and Utych (2023) document a tendency of urban and rural residents to discriminate against their place-based out-group. They find these biases in political and apolitical settings when respondents are asked to distribute resources between places or decide whom to hire among a set of hypothetical job applicants. In addition to that, Zumbrunn (2025) finds that Swiss citizens living in rural areas tend to be affectively polarised against urbanites, while urbanites do not seem to reciprocate this feeling. Furthermore, place-based identity and resentment tend to correlate with place-based affective polarisation in the Swiss context, increasing our confidence in the overall plausibility of our hypotheses. Yet, what both studies leave unexplored is the relationship between place-based affective polarisation and voting behaviour.

Against this backdrop, we suggest that place-based affective polarisation should be associated with voting for GAL and TAN parties. These parties take opposing positions along a transnational cleavage, which has developed over the last couple of decades in European politics (for an overview, see Marks et al., 2021). At its core, this divide concerns “the defense of national political, social and economic ways of life against external actors who penetrate the state by migrating, exchanging goods or exerting rule” (Hooghe & Marks, 2018, p. 110). Thereby, it comprises a fundamental opposition between GAL parties, who embrace open borders and international governance, and TAN parties, who stand for an outright rejection of these developments (e.g., Dassonneville et al., 2024; Hooghe & Marks, 2018; Hooghe et al., 2002; Hooghe et al., 2025).^3^

Crucially, there is first evidence suggesting clear associations between the GAL pole and urban voters on the one hand and the TAN pole and rural voters on the other. Based on comparative survey data from France, Germany, the United Kingdom, and Switzerland, Bornschier et al. (2022) find that urban residents are often regarded as new left voters, while rural residents tend to be seen as voting for either radical right or mainstream right parties. Using open-ended survey questions, Zollinger (2024a) further demonstrates that radical right voters in Switzerland often characterise their political in-groups as rural and out-groups as urban. In addition, Sczepanski (2024) finds that Austrians and Italians perceive rural individuals as more likely to support leaving the European Union, whereas urbanites are viewed as likely voters in favour of remaining. Similarly, in a conjoint experiment conducted in nine European countries, Hegewald (2024a) shows that urban and rural residents are perceived as fundamentally antagonistic social groups that take opposing positions on a multitude of politically charged dimensions. While typical ruralites are often viewed as Eurosceptic, anti-immigrant, working class, lower educated, and older, typical urbanites are perceived as Europhile, pro-immigrant, upper middle class, university educated, and younger.

From a group-based approach to partisanship, these perceptions of group alignments are key in connecting voters’ group memberships to their political choices. In short, group-based approaches to partisanship argue that voters compare the typical supporters of each party with their own group memberships and then choose the party whose typical supporters they believe most closely resemble themselves (e.g., Achen & Bartels, 2016; Green et al., 2004; Huddy et al., 2015; Kane et al., 2021; Miller et al., 1991; Miller & Wlezien, 1993; Wlezien & Miller, 1997). Thus, urban residents who like their urban in-group over their rural out-group should be more likely to vote for GAL parties as these are perceived as typically supported by urbanites. By contrast, following the same logic, ruralites who prefer their rural in-group over their urban out-group should be more supportive of TAN parties.

**H**_**4a**_: The stronger place-based affective polarisation among urban residents, the more likely they are to vote for GAL parties.
**H**_**4b**_: The stronger place-based affective polarisation among rural residents, the more likely they are to vote for TAN parties.

## Data and Methods

We test our hypotheses by drawing on original survey data from nine European countries (Czech Republic, Denmark, France, Germany, Greece, Hungary, Italy, Poland, and Spain). Our data were collected between February and April 2023 via open-access panels administered by the survey company Bilendi. Nationally representative quotas for age, gender, education, and NUTS-2 region were applied. About 1000 respondents were sampled in each country, resulting in a total sample size of 9114 respondents. For descriptive statistics and operationalisations of all variables employed in the analysis, see Tables A.1 and A.2 in the Appendix. For data collection periods per country, see Table A.3 in the Appendix.^4^

Our main measure of place-based affective polarisation relies on two thermometer ratings asking respondents to indicate how warm or cold they feel towards their respective place-based in-groups and out-groups. We code respondents’ in-groups and out-groups by preceding the thermometer questions with a self-classification item asking respondents whether they live in a “very rural”, “rather rural”, “rather urban” or “very urban” place. Respondents are coded as rural when they have indicated that they live in a “very rural” or “rather rural” place and urban when they have indicated otherwise. Using these two thermometer scores for urban and rural residents, we then follow the literature on affective partisan polarisation by forming a thermometer differential, where we subtract respondents’ out-group ratings from their in-group ratings (e.g., Iyengar et al., 2012, 2019). On this differential, positive values indicate higher place-based affective polarisation, where in-group affect exceeds out-group affect, while negative values mean the opposite.^5^

Place-based resentment is measured using a scale we adapted from Munis (2022). The scale consists of five items, asking urban (rural) respondents to indicate how much they agree with different statements about ruralites (urbanites) shortchanging their area for its fair share of resources, attention from policy-makers, and respect.^6^ Respondents answered each item on a 5-point Likert scale ranging from “strongly disagree” to “strongly agree”. From these answers, we then calculate an average scale (Cronbach’s α = 0.83).^7^

Our measure of place-based identity taps self-reported closeness to urban people for respondents who have indicated that they live in an urban place and closeness to rural people for respondents who have said that they live in a rural area. By way of that, this variable measures urban or rural in-group attachment depending on the stated group membership respondents have indicated on the urban-rural self-classification item. Both items were adapted from Bornschier et al. (2021) and answered by respondents on a scale from 1 (“not close at all”) to 10 (“very close”).^8^

We operationalise voting for GAL and TAN parties by relying on a question asking respondents which party they would vote for if there were an election for the country’s national parliament tomorrow. Taking this variable as a basis, we classify respondents’ vote choices with the help of expert survey data from the Chapel Hill Expert Survey (CHES). We rely on the most recent edition from the 2023 SPEED CHES wave (Hooghe, Marks, Bakker, et al., 2024). Specifically, we use the GAL-TAN item that ranks parties on an 11-point scale ranging from 0 (“libertarian/postmaterialist”) to 10 (“traditional/authoritarian”). We then code respondents’ vote intentions according to these party scores. More specifically, this means that if a person would vote, for instance, for the German Greens, in our data, we code this individual with the value the party receives on the CHES GAL-TAN item. Therefore, higher values on this variable represent a higher propensity for respondents to vote for TAN parties. By comparison, lower values stand for a higher propensity to vote for GAL parties.^9^ We opt for this coding of voting behaviour as this is a particularly suitable strategy to make individual electoral preferences comparable across our diverse set of countries.

To test H_1_, we show the distributions of place-based affective polarisation per country by self-classified urban-rural residence. We then run a series of simple unpaired t-tests to discern if the means between both groups are significantly different. We test H_2a_ to H_3b_ by splitting the sample into urban and rural sub-samples, again by relying on respondents’ urban-rural self-classifications. After this, we run two separate ordinary least squares (OLS) regressions with fixed effects at the country level, where we regress the thermometer differential on our measures of place-based resentment and identity.^10^ Lastly, we test H_4a_ and H_4b_ by regressing the GAL-TAN voting variable on an interaction term between the thermometer differential and a dummy variable indicating rural or urban residence according to respondents’ self-classifications.^11^ All models control for a range of potential confounders, including gender, age, education, income, migration background, and left-right self-placement.^12^

## Results

### Asymmetries in Place-Based Affective Polarisation

**Figure 1.**
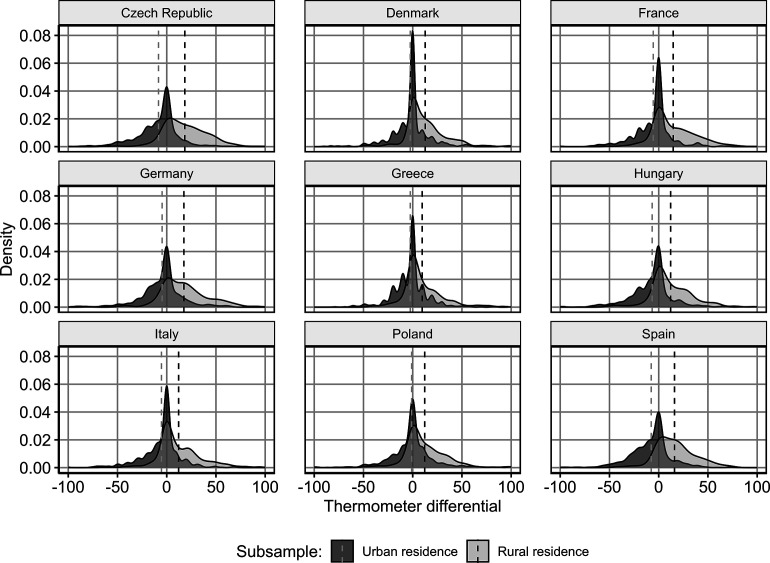
Distributions of place-based affective polarisation per country, by self-classified urban-rural residence. Kernel density plot. The thermometer differential indicates the difference between respondents’ in-group and out-group thermometer ratings. Positive values indicate higher place-based affective polarisation, where in-group ratings exceed out-group ratings, while negative values mean the opposite. The dashed lines indicate the mean values of the thermometer differential for urban and rural respondents respectively.

We start by describing the distribution of place-based affective polarisation for all nine countries surveyed in our study. To this end, Figure 1 plots the distributions of the thermometer differential per country by self-classified urban-rural residence. Clearly, place-based affective polarisation exists in all countries under investigation, albeit to different degrees. A significant number of respondents in each country exhibit a bias towards their respective urban or rural in-group, as indicated by the positive values of the differential. Overall, about 38.65% of respondents have a positive differential. This proportion is lowest in Hungary with 33.72% and highest in Germany with 46.79%. For many respondents in our data, we find a pronounced difference in the evaluations of place-based in-groups and out-groups, where the former is preferred over the latter. However, it is important to note that, for most respondents (61.35%), the thermometer differential is still either negative or zero, indicating that these individuals do not exhibit place-based affective polarisation. Furthermore, on average, place-based affective polarisation tends to be lower than affective partisan polarisation, as shown in Figure A.16 in the Appendix.^13^

We now turn to our first hypothesis on the asymmetry of place-based affective polarisation between urbanites and ruralites. As expected, place-based affective polarisation is more pronounced among rural residents. Within the rural sub-sample, 66.34% have a positive differential, while only 26.89% in the urban sub-sample are affectively polarised. Looking further at the dashed lines in Figure 1, which indicate the mean values of the thermometer differential for urban and rural residents, reveals that average place-based affective polarisation among ruralites considerably exceeds that among urbanites in all nine countries studied. Note, however, that the mean thermometer differentials for urban residents are negative, which indicates that urbanites are, on average, not affectively polarised. The gap in polarisation between both groups is largest in the Czech Republic, with a difference of 26.72 points, and smallest in Greece, with a difference of 12.08 points. A series of unpaired t-tests show that all of these differences are statistically significant at a *p <* 0.001 level (see Table A.15 in the Appendix). This supports our expectation of asymmetric levels of place-based affective polarisation between urban and rural residents (H_1_).

We have theorised that geographic sorting dynamics may help explain this asymmetry. While a comprehensive test of this mechanism would require detailed data on respondents’ residential histories, we now present first results to substantiate this argument with the data we have available. In addition to the urban-rural self-classification item introduced above, we also asked respondents to indicate whether they currently live in “a big city”, “the suburbs or outskirts of a big city”, “a town or small city”, “a country village”, or “a farm or home in the country-side”.^14^ Using the same response scale, our survey also includes a question about respondents’ residential biography, asking specifically about the type of place where they spent the majority of their childhood, up to the age of 15. Treating both variables as numeric, with higher values indicating more rural places and lower values indicating more urban places, we calculate a movement variable as the difference between the residential biography and current residence variables. Positive values indicate a move from a more rural to a more urban place, while negative values mean the opposite. Based on this movement variable we then create an urban move and a rural move indicator. The rural move variable is coded as 0 (“No”), when movement ≥ 0 and 1 (“Yes”) when movement *<* 0. Conversely, the urban move variable shows 0 (“No”), when movement ≤ 0 and 1 (“Yes”) when movement *>* 0.

**Figure 2.**
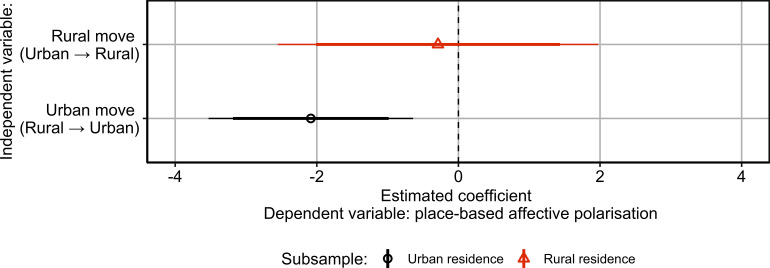
OLS regression results: place-based affective polarisation on movement indicators, by self-classified urban-rural residence. OLS regression coefficients with country fixed effects. Thick and thin lines are 95% and 99% confidence intervals, respectively. For full model results see Table A.16 in the Appendix.

Relying on these indicators as independent variables, Figure 2 presents the results of two OLS regressions, splitting the sample by respondents’ urban-rural self-classifications. We examine the relationship between place-based affective polarisation and the rural move variable in the rural sub-sample and the urban move variable in the urban sub-sample. Among ruralites, moving from a more urban to a more rural place has no effect on levels of place-based affective polarisation. However, urbanites who grew up in a more rural area and have since moved to a more urban location exhibit levels of place-based affective polarisation that are approximately 2.08 points lower. This finding remains robust when control variables are added (see Table A.17 in the Appendix). Overall, this speaks to the notion that divided loyalties mitigate place-based affective polarisation among urbanites and may help explain the asymmetry between rural and urban residents. Given that more individuals tend to move from the countryside to cities rather than the other way around, it seems plausible that urbanites are simply more divided in terms of their loyalties, resulting in lower levels of place-based affective polarisation compared to ruralites.

### Correlates of Place-Based Affective Polarisation

**Figure 3.**
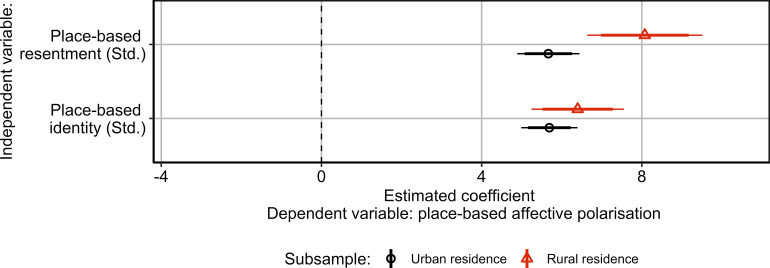
OLS regression results: place-based affective polarisation on place-based resentment and place-based identity, by self-classified urban-rural residence. OLS regression coefficients with country fixed effects. Thick and thin lines are 95% and 99% confidence intervals, respectively. Models control for gender, age, education, income, migration background, and left-right self-placement. For full model results see Table A.18 in the Appendix.

We now investigate our hypotheses on the correlations between place-based resentment, identity and place-based affective polarisation. Figure 3 presents the results of two OLS regressions, examining the correlates of place-based affective polarisation. We again split our sample with regard to respondents’ self-classifications of urban and rural residence, regressing the thermometer differential on our measures of place-based resentment and identity within each sub-sample. Full model results can be found in Table A.18 in the Appendix. Increased feelings of place-based resentment are associated with a stronger preference for one’s place-based in-group relative to the out-group among both urbanites and ruralites. With each standard deviation increase in place-based resentment, the mean level of the thermometer differential grows by 5.67 points among urbanites and 8.08 points among ruralites. This change corresponds to approximately a quarter of a standard deviation in the thermometer differential for urban residents and just over a third of a standard deviation for rural residents. Despite the moderate correlation between place-based resentment and affective polarisation (see Figure A.5 in the Appendix), these findings show that the two concepts are related in the theoretically expected direction. Some of the structural group conflicts and the associated threat perceptions in the place-based resentment items are associated with increased place-based affective polarisation. As expected, this association is more pronounced among ruralites.

Results for the relationship between place-based identity and affective polarisation are similar. Respondents with a stronger attachment to their urban or rural in-group tend to favour their in-group over their out-group. Among urban residents, the increase in place-based affective polarisation associated with a one standard deviation increase in place-based identity is about the same as that associated with a one standard deviation increase in place-based resentment. For rural residents, each standard deviation increase in place-based identity corresponds to a 6.40-point increase in the mean level of the thermometer differential. This is slightly more than a quarter of a standard deviation in place-based affective polarisation. The effect of place-based identity is again more pronounced among rural residents, but the asymmetry between urban and rural residents is smaller compared to place-based resentment.

The coefficients for both place-based resentment and identity are larger in the rural sub-sample. In Table A.19 in the Appendix, we estimate additional OLS regressions, where we interact place-based resentment and identity with respondents’ urban-rural self-classifications. These models show a more pronounced positive association between place-based resentment and identity with place-based affective polarisation among rural residents compared to urban residents. This result squares well with other studies documenting higher levels of place-based resentment and identity among ruralites (e.g., Borwein & Lucas, 2023; Haffert et al., 2024; Munis, 2022; Zumbrunn, 2024b).

Our findings on the correlates of place-based affective polarisation tend to hold across the different country contexts investigated. Table A.20 in the Appendix presents OLS regressions per country, regressing the thermometer differential on place-based resentment and identity. For reasons of low statistical power, we only show results without control variables whenever we look at the individual country samples. In all nine countries, place-based resentment and identity are strongly associated with an increase in place-based affective polarisation among both ruralites and urbanites. However, when it comes to differences in effect sizes between the urban and rural sub-samples, there seems to be some heterogeneity between countries. As shown in Table A.21 in the Appendix, the interactions between place-based identity and urban-rural self-classifications only reach conventional levels of statistical significance in Italy. Conversely, the interactions between urban-rural self-classifications and place-based resentment are statistically significant in all countries except for Hungary, Poland, and Spain.

**Figure 4.**
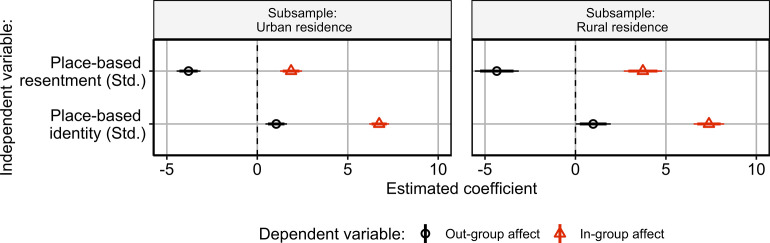
OLS regression results: in-group affect and out-group affect on place-based resentment and place-based identity, by self-classified urban-rural residence. OLS regression coefficients with country fixed effects. Thick and thin lines are 95% and 99% confidence intervals, respectively. Models control for gender, age, education, income, migration background, and left-right self-placement. For full model results see Table A.22 in the Appendix.

We delve deeper into the underlying mechanisms of our findings by examining the associations of place-based resentment and place-based identity with the constituent parts of the thermometer differential. Our theoretical arguments imply that we should see higher levels of place-based identity and resentment to decrease out-group affect, while simultaneously increasing in-group like. To test for this, Figure 4 summarises the results from four OLS regressions, where we regress either in-group or out-group affect on place-based resentment and place-based identity. We again split the sample by urban and rural residence following respondents’ self-classifications. Full model results can be found in Table A.22 in the Appendix. As we would expect, among urban and rural residents, higher levels of place-based resentment increase in-group affect and decrease out-group affect. Therefore, place-based resentment is associated with out-group negativity as well as in-group like.

By contrast, the positive coefficients for place-based identity in Figure 4 show that stronger attachment to one’s place-based in-group increases both in-group and out-group affect. This finding holds for both urbanites as well as ruralites and somewhat contradicts our theoretical arguments. However, that being said, the associations between place-based identity and in-group affect tend to be weaker than those with out-group affect. While a one standard deviation increase in place-based identity among urbanites (ruralites) is associated with a 6.74 (7.38) point increase in in-group affect, it only marginally increases out-group affect by 1.05 (0.98) points. In this regard, the association between place-based identity and place-based affective polarisation appears to be underpinned by considerable differences in the magnitude of the correlations with in-group and out-group affect.

Overall, our findings provide clear evidence supporting our expectation that stronger place-based resentment is correlated with higher levels of place-based affective polarisation (H_2a_). This relationship, in turn, tends to be more pronounced among rural residents (H_2b_). This suggests that the grievances and threat perceptions hard-wired into place-based resentment items capture a relevant part of affective polarisation of rural residents. For urbanites, these resentment items, however, contribute less to explaining affective polarisation. Our analysis of the relationship between place-based identity and place-based affective polarisation yields somewhat mixed results. Although we find a strong, positive association between place-based identity and the thermometer differential, we cannot conclude that this association is driven by a negative relationship with out-group affect, and a positive relationship with in-group affect. We, therefore, only count our findings as partial evidence for our expectation that strong place-based identity coincides with higher levels of place-based affective polarisation (H_3a_). Strong place-based identity polarises place-based affect but does not fuel out-group dislike. Furthermore, while we can show that the positive association between identity and affect is more pronounced among ruralites (H_3b_), this interaction does not consistently hold across the different country contexts under investigation. These findings make sense in light of the place-based resentment literature, as it might require place-based grievances to put urbanites and ruralites into an antagonistic conflict that generates out-group dislike. The existing place-based resentment items, however, seem more tuned towards capturing rural grievances, rather than urban ones.

### The Electoral Consequences of Place-Based Affective Polarisation

**Figure 5.**
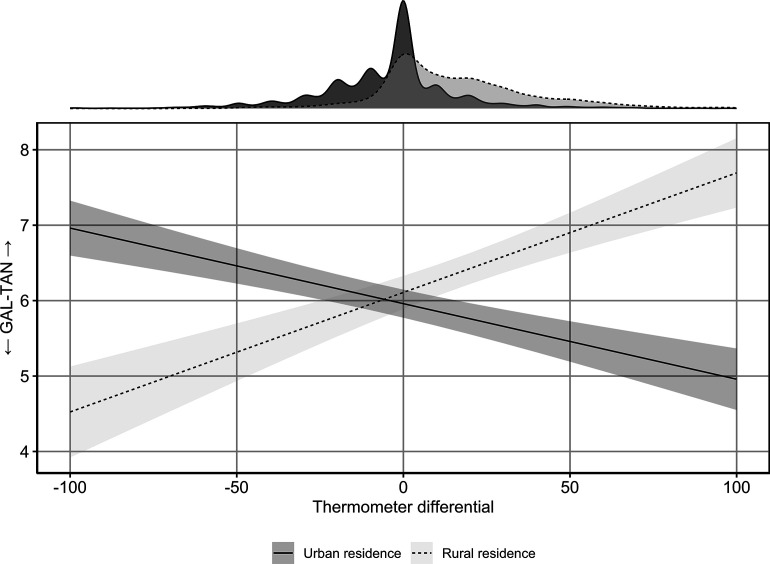
Predicted values of GAL-TAN voting variable by place-based affective polarisation, conditional on urban-rural self-classifications. Based on an OLS regression with country fixed effects. 95% confidence intervals displayed. The model controls for gender, age, education, income, migration background, and left-right self-placement. For full model results with a standardised version of the thermometer differential, see Table A.23 in the Appendix.

After we have examined the correlates of place-based affective polarisation, we will now focus on its electoral consequences. Figure 5 plots the predicted values of respondents’ vote intentions for GAL and TAN parties over different values of the thermometer differential, splitting the estimates by respondents’ urban-rural self-classifications. Full model results with a standardised version of the thermometer differential can be found in Table A.23 in the Appendix. The interaction term is statistically significant at a *p <* 0.001 level. Among urbanites, place-based affective polarisation increases voting for GAL parties. The more urban residents prefer their in-group over their out-group, the more they tend to vote for parties embracing transnationalism. By contrast, higher levels of place-based affective polarisation increase support for TAN parties among ruralites. As in-group bias among rural residents grows, so does their propensity to vote for TAN parties.

Substantially, our findings suggest that a one standard deviation increase in the thermometer differential results in a decrease in our GAL-TAN voting variable of –0.23 points among urban residents, and an increase of 0.36 points among rural residents. This shows that the effect of place-based affective polarisation is slightly more pronounced among ruralites, but also plays a role for urbanites. Overall, the size of these effects is small, given a standard deviation of 2.82 in our dependent variable. However, an increase by one standard deviation in place-based affective polarisation would, for example, still resemble a shift from the liberal FDP (GAL-TAN = 2.45) to the radical left party Die Linke (GAL-TAN = 2.73) within the German context. Furthermore, these marginal effects are calculated based on a statistical model with a set of demanding control variables, such as left-right self-placement. We therefore consider these results as lower-bound estimates.^15^

**Figure 6.**
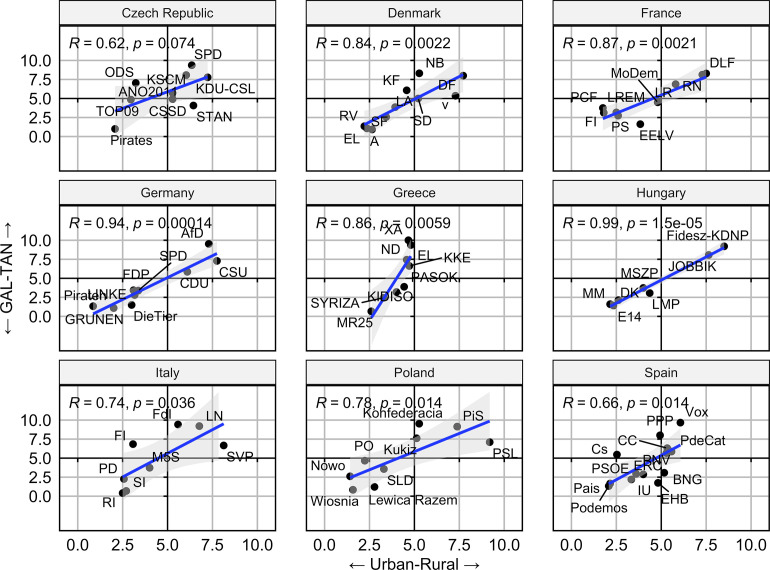
Correlations between urban-rural and GAL-TAN party positions, 2019. Pearson’s *R* correlation coefficients and statistical p-values for the bivariate relationship are depicted inside the plots. Data stem from the Chapel Hill Expert Survey trend file (Jolly et al., 2022). The GAL-TAN variable ranges from 0 (“libertarian/postmaterialist”) to 10 (“traditional/authoritarian”), and the urban-rural variable from 0 (“strongly supports urban interests”) to 10 (“strongly supports rural interests”). The solid line indicates a linear fit. The shaded area denotes a 95% confidence interval.

Our findings are robust across the different country contexts under investigation. As shown in Table A.25 in the Appendix, we observe a positive, statistically significant interaction term between the thermometer differential and respondents’ urban-rural self-classifications in all countries except Spain. We can only speculate why this is the case. To this end, Figure 6 plots the correlations between urban-rural and GAL-TAN party positions in all nine countries. The GAL-TAN party position variable is the same as before, and the urban-rural variable ranges from 0 (“strongly supports urban interests”) to 10 (“strongly supports rural interests”). Since urban-rural party positions are not available in the 2023 CHES wave, we have to rely on CHES data from 2019 for this analysis (Jolly et al., 2022). The bivariate correlations between urban-rural and GAL-TAN party positions are statistically significant at a *p <* 0.05 level, positive, and strong in all countries except for the Czech Republic. Overall, this illustrates well a pronounced overlap of parties’ orientations on the transnational cleavage and their positions regarding urban-rural interests. Parties at the GAL pole of the transnational divide tend to embrace urban issues, while parties more closely located to the TAN side are stronger proponents of rural interests.

Moreover, all countries seem to have at least one GAL party that is also a strong advocate of urban interests, as represented by each of the lower left quadrants in Figure 6. A similar picture emerges in the upper right quadrants, where most countries have at least one TAN party that strongly caters to ruralites. However, the party systems in Greece and Spain appear to lack a strong TAN proponent for rural interests. Whereas TAN parties in most of the countries under investigation marry a platform opposed to transnationalism with a pro-rural stance, this seems not to be the case in Greece and Spain. Crucially, when we replicate our analysis using the GAL-TAN item from the 2019 CHES wave as the basis for our dependent variable, we can observe that the interaction term between the thermometer differential and respondents’ urban-rural self-classifications also loses its statistical significance in Greece, dropping to a *p <* 0.1 level (see Table A.26 in the Appendix). Taken together, this might explain why we do not find a strong relationship between the thermometer differential and GAL-TAN voting in these countries.

**Figure 7.**
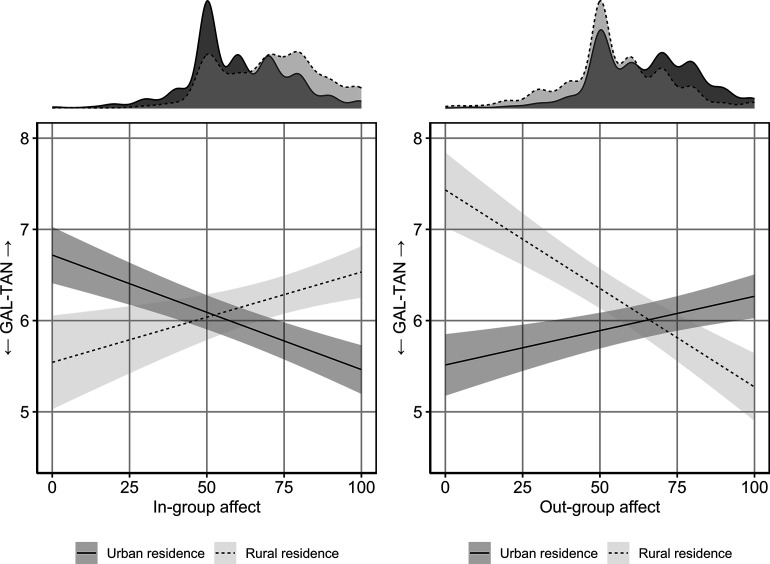
Predicted values of GAL-TAN voting variable by in-group affect and out-group affect, conditional on urban-rural self-classifications. Based on OLS regressions with country fixed effects. 95% confidence intervals displayed. Models control for gender, age, education, income, migration background, and left-right self-placement. For full model results with standardised versions of in-group and out-group affect, see Table A.27 in the Appendix.

Again, we dive deeper into our theoretical mechanism by investigating the effects of in-group and out-group like separately. Figure 7 plots the predicted values of respondents’ vote intentions for GAL and TAN parties over different values of in-group affect (left panel) and out-group affect (right panel), conditional on respondents’ urban-rural self-classifications. It illustrates why it is important to look at sentiments directed at place-based in-groups and out-groups in conjunction. Full model results with standardised versions of the in-group and out-group affect variables can be found in Table A.27 in the Appendix. Both interactions are included in the same model and are statistically significant at a *p <* 0.001 level. This already shows that even when controlling for in-group affect (out-group affect), out-group affect (in-group affect) retains its explanatory relevance for GAL-TAN voting. Furthermore, in-group and out-group affect are of different importance for structuring urbanites’ and ruralites’ respective voting intentions. Notably, the importance of in-group affect outweighs that of out-group affect among urban residents. A one standard deviation increase in in-group affect results in a –0.22 points decrease on the GAL-TAN voting variable, while the same change for out-group affect results in an increase of only 0.13 points. For rural residents, in turn, we see the opposite pattern. A one standard deviation increase in out-group affect corresponds to a –0.37 points decrease on the GAL-TAN variable, whereas a one standard deviation change in in-group affect only results in an increase of 0.17 points. Therefore, while both in-group and out-group affect seem to matter for GAL-TAN voting, the former tends to be more important for urbanites, while the latter appears to make a larger difference for ruralites. Crucially, we would have missed these nuances, if we would concentrate on in-group sentiments only.

In conclusion, our analysis of the electoral consequences of place-based affective polarisation provides strong evidence for our expectations that higher levels of place-based affective polarisation are associated with voting for GAL parties among urbanites (H_4a_), and support for TAN parties among ruralites (H_4b_). The more urbanites like their in-group over their out-group, the more they tend to vote for GAL parties. Conversely, as rural residents increasingly prefer their in-group over their out-group, the more they vote for TAN parties. We have also shown that the constituent parts of place-based affective polarisation, in-group affect and out-group affect, both matter for respondents’ vote choice. However, while in-group affect tends to make a larger difference for urbanites, out-group affect appears to be more important for ruralites. Theoretically, this finding lends further support to group-based explanations of the urban-rural divide, as voters seem to systematically relate their place-based group affect to their vote choice.

### Place-Based Affective Polarisation and Territorial Context

Our conceptualisation of place-based affective polarisation is heavily informed by insights from social identity theory (e.g., Tajfel, 1974; Tajfel, 1981; Tajfel & Turner, 1979; for an overview, see Brewer, 2019). Given this psychological approach, we rely on respondents’ self-classifications to tap into their place-based in-groups and out-groups. We thereby follow recent advice by Nemerever and Rogers (2021) to use subjective measures of urban-rural location when operationalising concepts relating to place as a social identity.

Nevertheless, respondents’ self-classifications correlate strongly with more objective definitions of urban-rural location, such as population density measured at the level of NUTS-3 regions (see Figure A.18 in the Appendix). In light of this, one might wonder to what extent territorial context shapes the role of place-based affective polarisation. Although we leave a systematic investigation of this to future research, we consider two ways in which territorial context could matter for our conclusions. First, place-based affective polarisation may primarily be driven by objective contextual conditions. For instance, in the case of affective partisan polarisation, existing research has shown that high unemployment and inequality are associated with greater polarisation (e.g., Gidron et al., 2020). Second, and building on the first point, if we find a strong correlation between place-based affective polarisation and contextual conditions, this may confound our results on its electoral consequences. As highlighted by the literature on place-based grievances reviewed above, factors such as persistent economic decline, regional inequalities, and the deterioration of public services play a crucial role in shaping territorial political divisions (e.g., Broz et al., 2021; Colantone & Stanig, 2018a; Cremaschi et al., 2024; Rodríguez-Pose, 2018). In this sense, territorial context could underlie both place-based affective polarisation and voting behaviour, potentially leading to a spurious correlation between the two.

We investigate how territorial context affects our results by exploiting the multilevel nature of our data, which clusters respondents in 742 NUTS-3 and 149 NUTS-2 regions.^16^ We start by estimating empty models with random intercepts at the NUTS-2 or NUTS-3 regional level to decompose the variance between regions and individuals. Full model results can be found in Table A.28 in the Appendix. Focusing on the intraclass correlation coefficients (*ICC*s) as a measure of the proportion of total variance that is attributable to differences between regions rather than individual-level differences, we find that variation at the NUTS-2 and NUTS-3 regional level is negligible for explaining variation in place-based affective polarisation. At the NUTS-2 (NUTS-3) level, an *ICC* of 0.022 (0.026) indicates that only 2.2% (2.6%) of the variance is between regions. Both values fall well below the commonly used threshold of *ICC > *0.05. However, this does not necessarily mean that contextual factors as a whole are unimportant in understanding place-based affective polarisation. Rather, it may suggest that NUTS regions are too heterogeneous, encompassing a mix of urban and rural areas, which could obscure contextual effects. Furthermore, contextual effects tend to vanish at higher levels of spatial aggregation (e.g., Dinesen & Sønderskov, 2015; Schraff & Hegewald, 2025). This points to the need for more granular geographic data, for instance, at the postcode- or neighbourhood-level, to better capture how contextual conditions might influence place-based affective polarisation.

By contrast, the *ICCs* in the empty models for GAL-TAN voting are considerably larger, with 0.062 at the NUTS-2 and 0.075 at the NUTS-3 level. In light of this, we fit multilevel regression models with random intercepts at the NUTS-2 regional level and fixed effects at the country level to model the contextual variation of GAL-TAN voting in our data. Full model results can be found in Table A.29 in the Appendix. We consecutively add variables measuring unemployment, population change, and quality of governance, all at the NUTS-2 regional level. Critically, our main finding on the electoral consequences of place-based affective polarisation is robust to the inclusion of these additional control variables. Higher levels of place-based affective polarisation still correlate with support for GAL parties among urbanites and support for TAN parties among ruralites.

## Conclusion

After decades of absence, the urban-rural divide has woken up. Urbanites and ruralites appear to continuously drift apart in the way they vote, the attitudes they hold, and the extent to which they support the political system more generally (e.g., Hegewald, 2024b; Huijsmans & Rodden, 2025; Jennings & Stoker, 2016; Lago, 2022; Maxwell, 2019, 2020; McKay et al., 2021; Mitsch et al., 2021; Rodden, 2019; Scala & Johnson, 2017; Stein et al., 2021; Taylor et al., 2024; Zumbrunn, 2024a). Existing explanations have highlighted the importance of geographic sorting, place-based grievances, identities, and resentment (e.g., Bornschier et al., 2021; Cramer, 2016; Gimpel & Hui, 2015; Huijsmans, 2023a, 2023b; Lunz Trujillo & Crowley, 2022; Maxwell, 2020; Munis, 2022; Rodríguez-Pose, 2018). However, the extent to which the urban-rural divide is also affectively polarised has only received limited attention so far.

Shedding light on this gap is critical for cleavage detection and promises to deepen our understanding of how place underpins political divisions. In response to this, we advance the concept of place-based affective polarisation, which we defined as the difference in affect between place-based in-groups and out-groups. Our analysis documents a substantive bias of respondents to prefer people from their own place over people from other places. These results fit with other studies that show how affective polarisation can also emerge around other social groups beyond partisanship (e.g., Hobolt et al., 2021; Van Noord et al., 2025). While place-based affective polarisation is present among urbanites and ruralites, it tends to be more pronounced among the latter. One possible mechanism underpinning this asymmetry might be found in geographic sorting dynamics. In line with this, we show that urbanites who grew up in a more rural area and moved to a more urban location exhibit considerably lower levels of place-based affective polarisation. However, a comprehensive test of this argument would require detailed data on respondents’ residential histories. We believe that this is a fruitful area for future research.

Furthermore, we find that higher levels of place-based affective polarisation are associated with strong feelings of place-based resentment and identity. Place-based resentment simultaneously lowers out-group affect and increases in-group affect. Conversely, we uncover a different pattern when it comes to place-based identity. Although place-based identity and place-based affective polarisation are positively correlated, stronger attachment to one’s place-based in-group does not result in more negative feelings towards one’s out-group. Nevertheless, we show that place-based resentment and identity are more strongly linked to place-based affective polarisation among rural residents.

Our analysis also reveals significant consequences of place-based affective polarisation for voting behaviour. The more urban residents like urbanites over ruralites, the higher their support for GAL parties. By contrast, increasing levels of place-based affective polarisation among ruralites coincide with voting for TAN parties. Both sentiments directed at place-based in-groups and out-groups are central here. Crucially, out-group affect retains its explanatory power when controlling for in-group affect. Moreover, we show that out-group affect is more important for vote choice among ruralites, while in-group affect matters more for urbanites. This nuances the literature on place-based identity, which predominantly focuses on individuals’ attachments to place-based in-groups (e.g., Bolet, 2021; Bornschier et al., 2021; Fitzgerald, 2018; Zollinger, 2024b).

Relying on comparative data has further allowed us to test the viability of our hypotheses in a diverse set of nine European countries. Given that many studies focus on single countries such as the United States (e.g., Cramer, 2016; Jacobs & Munis, 2023; Lunz Trujillo & Crowley, 2022; Lyons & Utych, 2023; Munis, 2022), Switzerland (e.g., Bornschier et al., 2021; Zollinger, 2024b; Zumbrunn, 2024a, 2024b, 2025), or the Netherlands (e.g., Huijsmans, 2023a, 2023b), our study is among the first to provide a more comparative picture. Although we show that most of our hypotheses hold in all nine countries under investigation, we also uncover some diverging patterns, particularly in Greece and Spain. Due to the relatively small number of countries, we could only speculate why this heterogeneity occurs. Clearly, more comparative research is necessary here to explain these differences more systematically.

Although we depart from a psychological understanding of place, further disentangling how contextual conditions shape place-based affective polarisation is also important. While our preliminary analysis at the level of NUTS-2 and NUTS-3 regions indicates that individual-level differences trump regional variation in place-based affective polarisation, further research is needed here. We suspect that our data are simply not fine-grained enough to meaningfully capture how territorial context affects place-based affective polarisation.

In this sense, further research should also investigate other possible explanations of place-based affective polarisation. Besides contextual factors, another critical driver could relate to the alignment of the urban-rural divide with other cleavages. According to Mason (2015, 2016, 2018), one crucial explanation for affective partisan polarisation in the United States can be found in the increasing alignment of partisanship with other salient social identities, specifically ideology, race, and religion. In this regard, when urbanites and ruralites perceive each other as very different social groups occupying opposing ends on various political cleavages, these perceptions of group alignment might also underpin place-based affective polarisation. Understanding whether and how several societal group conflicts align into an overarching universalism–particularism cleavage remains an important task for future research (Zollinger, 2024a).

Overall, our findings point to a pronounced affective basis that underpins the urban-rural divide in a number of European countries. Given that this is central to the development of a full-fledged cleavage (e.g., Bartolini & Mair, 1990; Borbáth et al., 2023), our study provides critical evidence suggesting that the urban-rural divide is indeed a relevant line of political conflict in Europe. Compared with other measures of urban-rural polarisation, we believe that our approach is particularly useful. Place-based affective polarisation addresses a key limitation of existing place-based resentment batteries, which hard-wire a limited set of grievances into their measurements (e.g., Claassen et al., 2025; Hegewald, 2024b; Huijsmans, 2023a, 2023b; Munis, 2022). On the one hand, this may lead researchers to overlook other structural conflicts that fuel animosities between urban and rural residents. On the other hand, priming respondents to think of the urban-rural divide in terms of specific grievances may lead to an overestimation of urban-rural polarisation. Remaining agnostic to the specific structural conflicts underpinning the urban-rural divide, we sidestep both of these problems, making our approach more conservative and flexible at the same time. Finding a pronounced degree of polarisation along the urban-rural divide without reverting to place-based grievances directly in our measure is a strong piece of evidence that divisions between cities and the countryside represent an important fault line in contemporary European politics.

## Supplemental Material

Supplemental Material - Is the Urban-Rural Divide Affectively Polarised? Comparative Evidence from Nine European Countries Supplemental Material for Is the Urban-Rural Divide Affectively Polarised? Comparative Evidence from Nine European Countries by Sven Hegewald and Dominik Schraff in Comparative Political Studies.

## Data Availability

Replication materials and code can be found at Hegewald and Schraff (2025).

## Notes

1. Besides the urban-rural divide, our definition of place-based affective polarisation could also be applied to the centre-periphery divide in European politics (e.g., Lipset & Rokkan, 1967; Ziblatt et al., 2024) or country-specific geographic fault lines such as between Eastern and Western Germany (e.g., Mau, 2024).

2. Besides these, Stephan and Stephan (2000) also highlight intergroup anxiety and negative stereotyping as potential sources of perceived threat.

3. A multitude of alternative labels, such as “integration-demarcation” (Kriesi et al., 2006), “cosmopolitan-communitarian” (Teney et al., 2014), “cosmopolitan-parochial” (De Vries, 2018), or “universalist-particularist” (Ha¨usermann & Kriesi, 2015), have been proposed to also describe this conflict.

4. Replication materials and code can be found at Hegewald and Schraff (2025).

5. Our main findings are robust to an alternative operationalisation of place-based affective polarisation that relies on trait ratings instead (for examples in the literature on affective partisan polarisation, see Garrett et al., 2014; Iyengar et al., 2012; Levendusky, 2018). The items we use are shown in Table A.4 in the Appendix, asking respondents to rate their respective placed-based in-groups and out-groups according to three positive characteristics (intelligent, open-minded and honest) and two negative characteristics (selfish and hypocritical). Each item was answered on a 5-point Likert scale ranging from “strongly disagree” to “strongly agree”. After reverse coding the negative items (selfish and hypocritical), we calculate average scales for in-group affect (Cronbach’s α = 0.74) and out-group affect (Cronbach’s α = 0.66). We then form a trait-rating differential by subtracting respondents’ out-group affect from their in-group affect. For distributions of the trait- rating differential per country, see Figure A.1 in the Appendix. For a replication of our main results, see Figures A.2 and A.3, and Tables A.5 and A.6 in the Appendix.

6. For distributions of place-based resentment per country, and the items we use, see Figure A.4 and Table A.7 in the Appendix.

7. For a correlation matrix between the different place-based resentment items, the place-based resentment scale, and place-based affective polarisation, see Figure A.5 in the Appendix. The bivariate correlations between the place-based resentment and affective polarisation items are weak to moderate, suggesting that these are distinct concepts.

8. For distributions of place-based identity per country, see Figure A.6 in the Appendix. We assess the robustness of our findings by replicating our main results with a variable measuring respondents’ attachment to their place of residence instead. For distributions of attachment to place of residence per country, see Figure A.7 in the Appendix. For the replication of our results, see Figure A.8 and Table A.8 in the Appendix. While the results in the rural sample are robust to this alternative operationalisation of place-based identity, the significance level of the coefficient in the urban sample seems to depend on the control variables included in the model.

9. For distributions of the GAL-TAN voting variable per country, see Figure A.9 in the Appendix. We replicate our main results by estimating additional models focusing on vote intention for radical right and green parties as two alternative dependent variables. These dummy variables are coded as 1 if respondents indicated they would vote for a green/radical right party and 0 if respondents indicated that they would vote for any other party (except the “other” category). We code green and radical right parties according to the party family variable in the CHES. For a replication of our results, see Figure A.10, and Tables A.9 and A.10 in the Appendix. Our substantive findings remain the same.

10. As a robustness check, we also split the sample using the full urban-rural self-classification item (see Figure A.11 and Table A.11 in the Appendix). Our substantive findings remain the same.

11. We assess the robustness of our findings by also estimating an interaction between the thermometer differential and the full urban-rural self-classification item (see Figure A.12 and Table A.12 in the Appendix). Our substantive findings remain the same. Following Hainmueller et al. (2019), we also test the linearity assumption of the interaction by using a simple binning estimator (see Figure A.13 in the Appendix).

12. Our main findings are robust to the inclusion of immigration attitudes as a substitute control variable for left-right self-placement. For a replication of our results, see Figures A.14 and A.15, and Tables A.13 and A.14 in the Appendix.

13. Furthermore, the correlation between place-based affective polarisation and affective partisan polarisation can be considered weak (Pearson’s *R* = 0.14).

14. Figure A.17 in the Appendix compares this measure with the urban-rural self-classification item, revealing a strong correlation between the two.

15. Comparing the effect sizes of place-based affective polarisation and resentment is also informative. In Table A.24 in the Appendix, we estimate additional OLS regressions, including interaction terms between place-based resentment and the urban-rural self-classification dummy as an additional control. In these specifications, the effect of place-based affective polarisation among urbanites (–0.20) and ruralites (0.31) is considerably larger than that of resentment among urban (–0.09) and rural residents (0.20). Furthermore, the significance level of the resentment interaction term seems to depend on the control variables included in the model, while the place-based affective polarisation interaction remains statistically significant across all model specifications.

16. The average number of respondents per NUTS-3 (NUTS-2) region is 12.28 (61.17).
