# Supplementary material for: Is the Urban-Rural Divide Affectively Polarised? Comparative Evidence from Nine European Countries
Source: Comp Polit Stud. 2025 Aug 29;59(10):2159–200. doi: 10.1177/00104140251369317 (PMC13427094; doi:10.1177/00104140251369317)
Supplement: Supplemental Material - Is the Urban-Rural Divide Affectively Polarised? Comparative Evidence from Nine European Countries [file sj-pdf-1-cps-10.1177_00104140251369317.pdf]

Online Appendix: "Is the Urban-Rural Divide Affectively Polarised? Comparative Evidence From Nine European Countries"

Sven Hegewald*   Dominik Schraff†

*Postdoctoral Researcher, Center for Comparative and International Studies, ETH Zurich, Zurich, Switzerland.
†Associate Professor, Department of Politics and Society, Aalborg University, Aalborg, Denmark.

List of Figures

A.1 Distributions of place-based affective polarisation per country, by self-classified urban-rural residence (trait-rating differential). 16
A.2 OLS regression results: place-based affective polarisation on place-based resentment and place-based identity, by self-classified urban-rural residence (trait-rating differential). 17
A.3 Predicted values of GAL-TAN voting variable by place-based affective polarisation, conditional on self-classified urban-rural residence (trait-rating differential). 19
A.4 Distributions of place-based resentment per country, by self-classified urban-rural residence. 21
A.5 Correlations between place-based resentment and place-based affective polarisation, by self-classified urban-rural residence. 22
A.6 Distributions of place-based identity per country, by self-classified urban-rural residence. 23
A.7 Distributions of attachment to place of residence per country, by self-classified urban-rural residence. 24
A.8 OLS regression results: place-based affective polarisation on place-based resentment and attachment to place of residence, by self-classified urban-rural residence. 25
A.9 Distributions of GAL-TAN voting per country. 27
A.10 Predicted probabilities of radical right and green voting by place-based affective polarisation, conditional on self-classified urban-rural residence. 28
A.11 OLS regression results: place-based affective polarisation on place-based resentment and place-based identity, by self-classified urban-rural residence (full variable). 31
A.12 Predicted values of GAL-TAN voting variable by place-based affective polarisation, conditional on urban-rural self-classifications (full variable). 33
A.13 Marginal effects of self-classified rural residence/urban residence on GAL-TAN voting, conditional on place-based affective polarisation. 35
A.14 OLS regression results: place-based affective polarisation on place-based resentment and place-based identity, by self-classified urban-rural residence (controlling for immigration attitudes). 35
A.15 Predicted values of GAL-TAN voting variable by place-based affective polarisation, conditional on urban-rural self-classifications (controlling for immigration attitudes). 37
A.16 Distributions of place-based affective polarisation and affective partisan polarisation per country. 39
A.17 Urban-rural self-classification variables compared. 40
A.18 Urban-rural self-classification and population density. 51

List of Tables

A.1 Summary statistics of all variables. 5
A.2 Details on measurement of variables. 7
A.3 Data collection periods per country. 15
A.4 Place-based affective polarisation trait ratings. 15
A.5 OLS regression results: place-based affective polarisation on place-based resentment and place-based identity, by self-classified urban-rural residence (trait-rating differential). 18
A.6 OLS regression results: GAL-TAN voting on place-based affective polarisation, conditional on urban-rural self-classifications (trait-rating differential). . . 20
A.7 Place-based resentment items. 21
A.8 OLS regression results: place-based affective polarisation on place-based resentment and attachment to place of residence, by self-classified urban-rural residence. 26
A.9 OLS regression results: radical right and green voting on place-based affective polarisation. 29
A.10 Logistic regression results: radical right and green voting on place-based affective polarisation. 30
A.11 OLS regression results: place-based affective polarisation on place-based resentment and place-based identity, by self-classified urban-rural residence (full variable). 32
A.12 OLS regression results: GAL-TAN voting on place-based affective polarisation, conditional on self-classified urban-rural residence (full variable). 34
A.13 OLS regression results: place-based affective polarisation on place-based resentment and place-based identity, by self-classified urban-rural residence (controlling for immigration attitudes). 36
A.14 OLS regression results: GAL-TAN voting on place-based affective polarisation, conditional on self-classified urban-rural residence (controlling for immigration attitudes). 38
A.15 Results of unpaired t-tests comparing thermometer differential scores between self-classified rural and urban residents per country (one-tailed). 38
A.16 OLS regression results: place-based affective polarisation on movement indicators, by self-classified urban-rural residence. 40
A.17 OLS regression results: place-based affective polarisation on movement indicators, by self-classified urban-rural residence (with control variables). 41
A.18 OLS regression results: place-based affective polarisation on place-based resentment and place-based identity, by self-classified urban-rural residence. . . . 42
A.19 OLS regression results: place-based affective polarisation on place-based resentment and place-based identity, conditional on self-classified urban-rural residence. 43
A.20 OLS regression results: place-based affective polarisation on place-based resentment and place-based identity, by self-classified urban-rural residence (per country). 44
A.21 OLS regression results: place-based affective polarisation on place-based resentment and place-based identity, conditional on self-classified urban-rural residence (per country). 45

A.22 OLS regression results: in-group affect and out-group affect on place-based resentment and place-based identity, by self-classified urban-rural residence. . . 46
A.23 OLS regression results: GAL-TAN voting on place-based affective polarisation, conditional on self-classified urban-rural residence. 47
A.24 OLS regression results: GAL-TAN voting on place-based affective polarisation, conditional on self-classified urban-rural residence (including interaction with place-based resentment). 48
A.25 OLS regression results: GAL-TAN voting on place-based affective polarisation, conditional on self-classified urban-rural residence (per country). 49
A.26 OLS regression results: GAL-TAN voting on place-based affective polarisation, conditional on self-classified urban-rural residence (CHES 2019 data; per country). 50
A.27 OLS regression results: GAL-TAN voting on in-group affect and out-group affect, conditional on self-classified urban-rural residence. 52
A.28 Multilevel regression results: empty models of place-based affective polarisation and GAL-TAN voting. 52
A.29 Multilevel regression results: GAL-TAN voting on place-based affective polarisation, conditional on urban-rural self-classifications (random intercepts for NUTS-2 region). 53

Table A.1: Summary statistics of all variables.

Statistic	N	Mean	St. Dev.	Min	Max
Age	9,114	48.53	15.88	18	93
Age (Std.)	9,114	0.00	1.00	−1.92	2.80
Gender	9,114	0.52	0.50	0	1
Education	9,114	0.30	0.46	0	1
Income (Deciles)	7,412	5.50	2.87	1	10
GAL-TAN vote	6,430	5.49	2.82	0.57	10.00
Radical right vote	6,430	0.29	0.46	0	1
Green vote	6,430	0.08	0.27	0	1
Thermometer differential	9,114	0.89	22.51	−100	100
Thermometer differential (Std.)	9,114	0.00	1.00	−4.48	4.40
Thermometer ratings in-group	9,114	61.49	17.19	0	100
Thermometer ratings in-group (Std.)	9,114	0.00	1.00	−3.58	2.24
Thermometer ratings out-group	9,114	60.60	17.17	0	100
Thermometer ratings out-group (Std.)	9,114	0.00	1.00	−3.53	2.29
Trait-rating differential	9,114	−0.12	0.90	−4.00	4.00
Trait-rating differential (Std.)	9,114	0.00	1.00	−4.29	4.57
Trait ratings in-group	9,114	3.10	0.65	1.00	5.00
Trait ratings out-group	9,114	3.22	0.58	1.00	5.00
Intelligence rating (in-group)	9,114	3.36	0.77	1	5
Open-mindedness rating (in-group)	9,114	3.43	0.94	1	5
Honesty rating (in-group)	9,114	3.10	0.97	1	5
Selfishness rating (in-group; reversed)	9,114	2.74	0.98	1	5
Hypocrisy rating (in-group; reversed)	9,114	2.87	0.98	1	5
Intelligence rating (out-group)	9,114	3.35	0.75	1	5

Continued on next page

Continued from previous page

Statistic	N	Mean	St. Dev.	Min	Max
Open-mindedness rating (out-group)	9,114	3.21	0.98	1	5
Honesty rating (out-group)	9,114	3.41	0.88	1	5
Selfishness rating (out-group; reversed)	9,114	3.05	0.91	1	5
Hypocrisy rating (out-group; reversed)	9,114	3.10	0.89	1	5
Place-based resentment	9,114	2.92	0.85	1.00	5.00
Place-based resentment (Std.)	9,114	0.00	1.00	−2.26	2.45
Economic	9,114	2.95	0.98	1	5
Representation (A)	9,114	2.95	1.11	1	5
Representation (B)	9,114	2.86	1.18	1	5
Culture (A)	9,114	3.02	1.09	1	5
Culture (B)	9,114	2.83	1.15	1	5
Place-based identity	9,114	7.06	2.04	1	10
Place-based identity (Std.)	9,114	0.00	1.00	−2.97	1.44
Urban identity (urban sample only)	6,396	6.95	2.04	1	10
Rural identity (rural sample only)	2,718	7.32	2.02	1	10
Attachment to place of residence	9,114	7.77	2.43	0	10
Attachment to place of residence (Std.)	9,114	0.00	1.00	−3.19	0.92
Left-right	9,114	5.10	2.40	0	10
Left-right (Std.)	9,114	0.00	1.00	−2.13	2.05
Urban-rural (full variable)	9,114	1.02	1.00	0	3
Urban-rural (binary)	9,114	0.30	0.46	0	1
Migration background	9,097	0.14	0.35	0	1
Immigration attitudes	9,114	4.51	2.65	0	10
Immigration attitudes (Std.)	9,114	0.00	1.00	−1.70	2.07
Residential biography	9,114	2.64	1.20	1	5

Continued on next page

Continued from previous page

Statistic	N	Mean	St. Dev.	Min	Max
Current residence	9,114	2.53	1.16	1	5
Movement	9,114	0.11	1.21	−4	4
Urban move	9,114	0.24	0.43	0	1
Rural move	9,114	0.18	0.38	0	1
Population density	9,089	1,023.40	2,293.16	8.70	20,897.10
Regional unemployment	9,071	6.27	4.01	1.70	26.60
Regional unemployment (Std.)	9,071	0.00	1.00	−1.14	5.06
Population Δ	9,114	4.64	12.23	−22.40	62.20
Population Δ (Std.)	9,114	0.00	1.00	−2.21	4.71
EQI	8,100	−0.27	0.98	−2.06	2.10

Table A.2: Details on measurement of variables.

Variable	Description
Age (Std.)	Self-reported age in years. Standardised.
Gender	Self-reported gender of respondent. 0 (“Male”), 1 (“Female”). 11 respondents who indicated “Non-binary” were dropped from the sample.
Education	“Have you completed a degree of higher education at a university or a similar institution (e.g., a Bachelor’s, Master’s, or PhD degree)?” Answer categories 0 (“No”) = Low, 1 (“Yes”) = High.

Continued on next page

Continued from previous page

Variable	Description
Income (Deciles)	Monthly net income of respondent's household in local currency. Measured in deciles for each country. In case of a tie, respondents were randomly assigned to either the lower or the higher bin.
GAL-TAN vote	"If there was an election for [country's national parliament] tomorrow, for which party would you vote?" Country-specific answer categories as well as an "Other" and "No answer" option. Classified according to GAL-TAN variable from the Chapel Hill Expert Survey using the 2023 SPEED CHES - Ukraine wave (Hooghe et al., 2024). Respondents receive the GAL-TAN score of the party they would vote for. 848 respondents who indicated "Other" and 1,800 respondents who chose "No answer" were coded as missing. We also coded 36 respondents as missing who would vote for a party that was not included in the CHES.

Continued on next page

Continued from previous page

Variable	Description
Radical right vote	"If there was an election for [country's national parliament] tomorrow, for which party would you vote?" Country-specific answer categories as well as an "Other" and "No answer" option. Classified according to the party family variable from the Chapel Hill Expert Survey using the 2023 SPEED CHES - Ukraine wave (Hooghe et al., 2024). Respondents who would vote for a "TAN" party are coded as 1, all other respondents are coded as 0. 848 respondents who indicated "Other" and 1,800 respondents who chose "No answer" were coded as missing. We also coded 36 respondents as missing who would vote for a party that was not included in the CHES.
Green vote	"If there was an election for [country's national parliament] tomorrow, for which party would you vote?" Country-specific answer categories as well as an "Other" and "No answer" option. Classified according to the party family variable from the Chapel Hill Expert Survey using the 2023 SPEED CHES - Ukraine wave (Hooghe et al., 2024). Respondents who would vote for a "Green" party are coded as 1, all other respondents are coded as 0. 848 respondents who indicated "Other" and 1,800 respondents who chose "No answer" were coded as missing. We also coded 36 respondents as missing who would vote for a party that was not included in the CHES.

Continued on next page

Continued from previous page

Variable	Description
Thermometer differential (Std.)	Difference between in-group thermometer ratings and out-group thermometer ratings. Positive values indicate higher place-based affective polarisation, where in-group affect exceeds out-group affect, while negative values mean the opposite. Standardised.
Thermometer ratings in-group (Std.)	"Previously, we have asked you about your place of residence. We now want you to judge people from other areas on a so-called 'feeling thermometer'. Scores between 50 and 100 mean that you have positive and warm feelings towards people from a certain area. Scores between 0 and 50 mean you feel cold and negative about the group. A score of 50 means you feel neither warm nor cold about people from the area. How do you feel about. . . ?" - ..."people from [in]". If respondents see themselves as living in a very rural/rather rural place: [in] = rural areas. If respondents see themselves as living in a very urban/rather urban place: [in] = urban areas. Standardised.

Continued on next page

Continued from previous page

Variable	Description
Thermometer ratings out-group (Std.)	"Previously, we have asked you about your place of residence. We now want you to judge people from other areas on a so-called 'feeling thermometer'. Scores between 50 and 100 mean that you have positive and warm feelings towards people from a certain area. Scores between 0 and 50 mean you feel cold and negative about the group. A score of 50 means you feel neither warm nor cold about people from the area. How do you feel about. . . ?" - ..."people from [out]". If respondents see themselves as living in a very rural/rather rural place: [out] = urban areas; If respondents see themselves as living in a very urban/rather urban place: [out] = rural areas. Standardised.
Trait-rating differential (Std.)	Difference between in-group trait ratings and out-group trait ratings. Positive values indicate higher place-based affective polarisation, where in-group affect exceeds out-group affect, while negative values mean the opposite. Standardised.
Trait ratings in-group	Average scale from in-group trait ratings. For items see Table A.4. Selfishness and hypocrisy ratings are reverse coded.
Trait ratings out-group	Average scale from out-group trait ratings. For items see Table A.4. Selfishness and hypocrisy ratings are reverse coded.

Continued on next page

Continued from previous page

Variable	Description
Place-based resentment (Std.)	Average scale from place-based resentment items. For items see Table A.7 (adapted from Munis, 2022). Standardised.
Place-based identity (Std.)	"Of the following groups, how close do you feel towards them? By 'close' we mean people who are most like you in terms of their ideas, interests, and feelings." - "People in urban areas" / "People in rural areas". Answer categories range from 1 ("Not close at all") to 10 ("Very close"). Adapted from Bornschier (2021). If respondents see themselves as living in a very rural/rather rural place, respondents receive their score on the rural closeness item. If respondents see themselves as living in a very urban/rather urban place, respondents receive their score on the urban closeness item. Standardised.
Attachment to place of residence (Std.)	"On a scale of 0 to 10 (where 0 means 'not attached at all', and 10 means 'strongly attached'), how attached do you feel with respect to ...?" – "Your place of residence" Answer categories from 0 ("Not attached at all") to 10 ("Strongly attached"). Standardised.
Left-right (Std.)	"Many people talk of 'left' and 'right' when describing different political views. Below we present you a scale from left (0) to right (10). Thinking about your own political views, where would you place yourself on this scale?" Answer categories range from 0 ("Left") to 10 ("Right"). Standardised.

Continued on next page

Continued from previous page

Variable	Description
Urban-rural (full variable)	"Do you live in an urban or rural area?" Answer categories 0 ("Very rural"), 1 ("Rather rural"), 2 ("Rather urban"), 3 ("Very urban"). Reverse coded so it ranges from 0 ("Very urban") to 3 ("Very rural").
Urban-rural (binary variable)	Based on urban-rural (full variable). 0 ("Rather urban" and "Very urban") = Urban residence, 1 ("Rather rural" and "Very rural") = Rural residence.
Migration background	"Were you and/or one of your parents born outside [respondent's country]?" Answer categories 0 ("No"), 1 ("Yes"). 17 respondents who indicated "Don't know" were coded as missing.
Immigration attitudes (Std.)	"Do immigrants make [respondent's country] a better or worse place to live?" Answer categories range from 0 ("A worse place") to 10 ("A better place"). Standardised.
Residential biography	"In what kind of place did you spend the greater part of your childhood, until you were 15 years old?" Answer categories 1 ("A big city"), 2 ("The suburbs or outskirts of a big city"), 3 ("A town or small city"), 4 ("A country village"), 5 ("A farm or home in the countryside").
Current residence	"Of the following categories, which describes best where you live?" Answer categories 1 ("A big city"), 2 ("The suburbs or outskirts of a big city"), 3 ("A town or small city"), 4 ("A country village"), 5 ("A farm or home in the countryside").

Continued on next page

Continued from previous page

Variable	Description
Movement	Difference between residential biography and current residence. Positive values indicate a move from a more rural to a more urban place. Negative values indicate a move from a more urban to to a more rural place.
Urban move	Based on movement variable. Coded as 0 (“No”), when movement ≤ 0 and 1 (“Yes”) when movement > 0.
Rural move	Based on movement variable. Coded as 0 (“No”), when movement ≥ 0 and 1 (“Yes”) when movement < 0.
Population density	Population density by NUTS-3 region in 2021. Data stem from Eurostat. Missing data for regions ITG2D, ITG2E, ITG2F, ITG2G, and ITG2H. Data were accessed in June 2025.
Regional unemployment	Total unemployment rate by NUTS-2 region across all ISCED 2011 levels in 2023. Data stem from Eurostat. Missing data for regions PL43, DE22, and DEB2. Data were accessed in June 2025. Standardised.
Population Δ	Population change by NUTS-2 region in 2022. Crude rate of total population change. Data stem from Eurostat. Missing data for regions PL43, DE22, and DEB2. Data were accessed in June 2025. Standardised.
EQI	European Quality of Government Index by NUTS-2 region in 2024. Data stem from Charron et al., (2024). Missing data for Germany and region ES64.

Table A.3: Data collection periods per country.

Czech Republic	07 February 2023 – 13 April 2023
Denmark	07 February 2023 – 19 March 2023
France	08 February 2023 – 14 March 2023
Germany	08 February 2023 – 13 March 2023
Greece	23 February 2023 – 01 April 2023
Hungary	09 February 2023 – 23 March 2023
Italy	24 February 2023 – 22 March 2023
Poland	09 February 2023 – 27 March 2023
Spain	17 February 2023 – 23 March 2023

Table A.4: Place-based affective polarisation trait ratings.

People from [in] are...	People from [out] are...
a. Intelligent	a. Intelligent
b. Open minded	b. Open minded
c. Honest	c. Honest
d. Selfish	d. Selfish
e. Hypocritical	e. Hypocritical

Note: If respondents see themselves as living in a very rural/rural place: [in] = rural areas; [out] = urban areas. If respondents see themselves as living in a very urban/urban place: [in] = urban areas; [out] = rural areas.

Figure A.1: Distributions of place-based affective polarisation per country, by self-classified urban-rural residence (trait-rating differential).

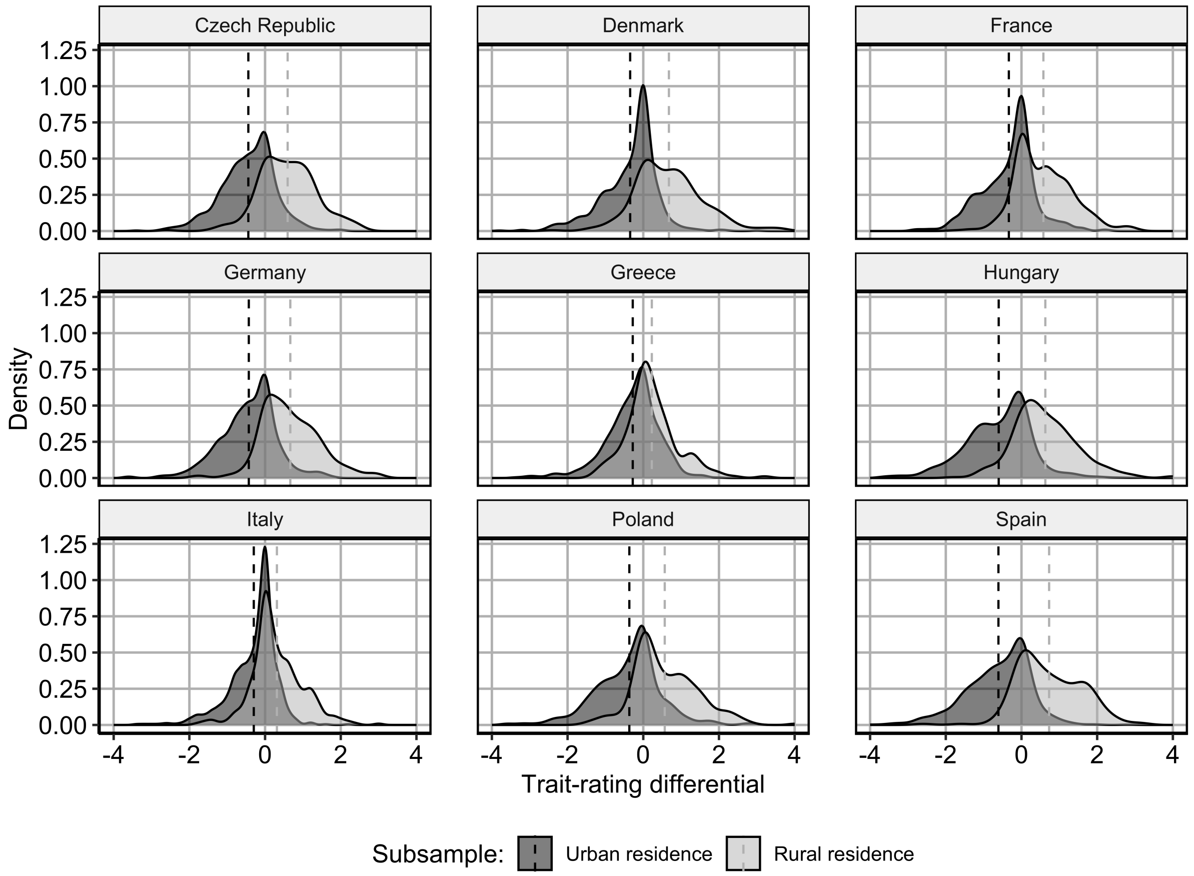

Note: Kernel density plot. The trait-rating differential indicates the difference between respondents' in-group and out-group trait ratings. Positive values indicate higher place-based affective polarisation, where in-group ratings exceed out-group ratings, while negative values mean the opposite. The dashed lines indicate the mean values of the trait-rating differential for urban and rural respondents respectively.

Figure A.2: OLS regression results: place-based affective polarisation on place-based resentment and place-based identity, by self-classified urban-rural residence (trait-rating differential).

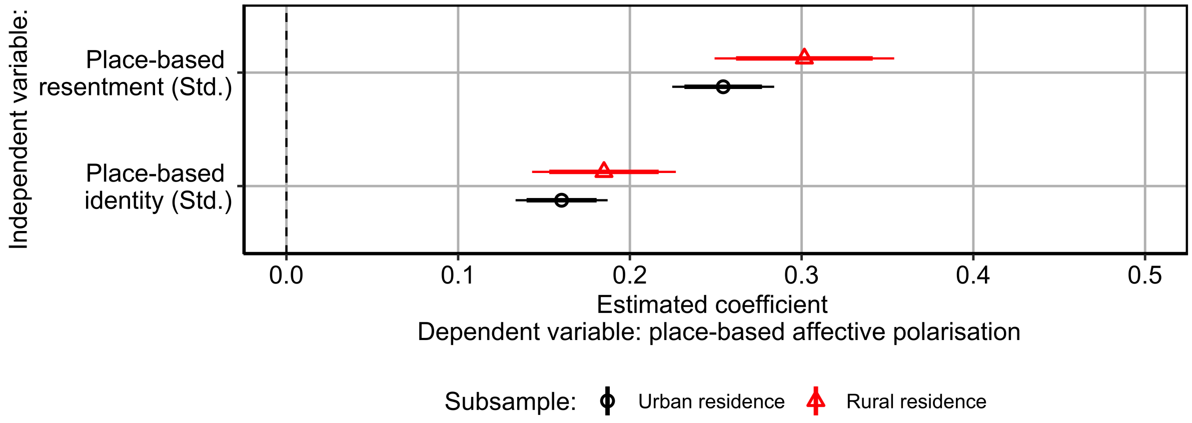

Note: OLS regression coefficients with country fixed effects. Thick and thin lines are 95% and 99% confidence intervals, respectively. Models control for gender, age, education, income, migration background, and left-right self-placement. For full model results see Table A.5 in Appendix B.

Table A.5: OLS regression results: place-based affective polarisation on place-based resentment and place-based identity, by self-classified urban-rural residence (trait-rating differential).

	Urban sample			Rural sample		
	(1)	(2)	(3)	(4)	(5)	(6)
Place-based resentment (Std.)	0.251***	0.252***	0.254***	0.288***	0.300***	0.302***
	(0.011)	(0.012)	(0.012)	(0.018)	(0.020)	(0.020)
Place-based identity (Std.)	0.155***	0.156***	0.160***	0.176***	0.190***	0.185***
	(0.009)	(0.010)	(0.010)	(0.014)	(0.016)	(0.016)
Gender (b.=male)		0.043*	0.030		−0.135***	−0.133***
		(0.021)	(0.020)		(0.031)	(0.031)
Age (Std.)		0.010	0.010		−0.039*	−0.038*
		(0.010)	(0.010)		(0.017)	(0.017)
Education (b.=low)		0.061**	0.053*		0.033	0.036
		(0.022)	(0.022)		(0.038)	(0.038)
Income (Deciles)		−0.002	0.000		0.003	0.003
		(0.004)	(0.004)		(0.005)	(0.005)
Migration background (b.=no)		−0.004	−0.009		−0.152**	−0.151**
		(0.029)	(0.029)		(0.046)	(0.046)
Left-right (Std.)			−0.097***			0.035*
			(0.010)			(0.015)
Constant	−0.282***	−0.297***	−0.286***	0.297***	0.344***	0.348***
	(0.027)	(0.038)	(0.038)	(0.044)	(0.061)	(0.061)
Country fixed effects	Yes	Yes	Yes	Yes	Yes	Yes
Num.Obs.	6,396	5,204	5,204	2,718	2,199	2,199
R2	0.149	0.157	0.172	0.187	0.208	0.210
R2 Adj.	0.148	0.154	0.170	0.184	0.202	0.204

+ $p < 0.1$, * $p < 0.05$, ** $p < 0.01$, *** $p < 0.001$

Figure A.3: Predicted values of GAL-TAN voting variable by place-based affective polarisation, conditional on self-classified urban-rural residence (trait-rating differential).

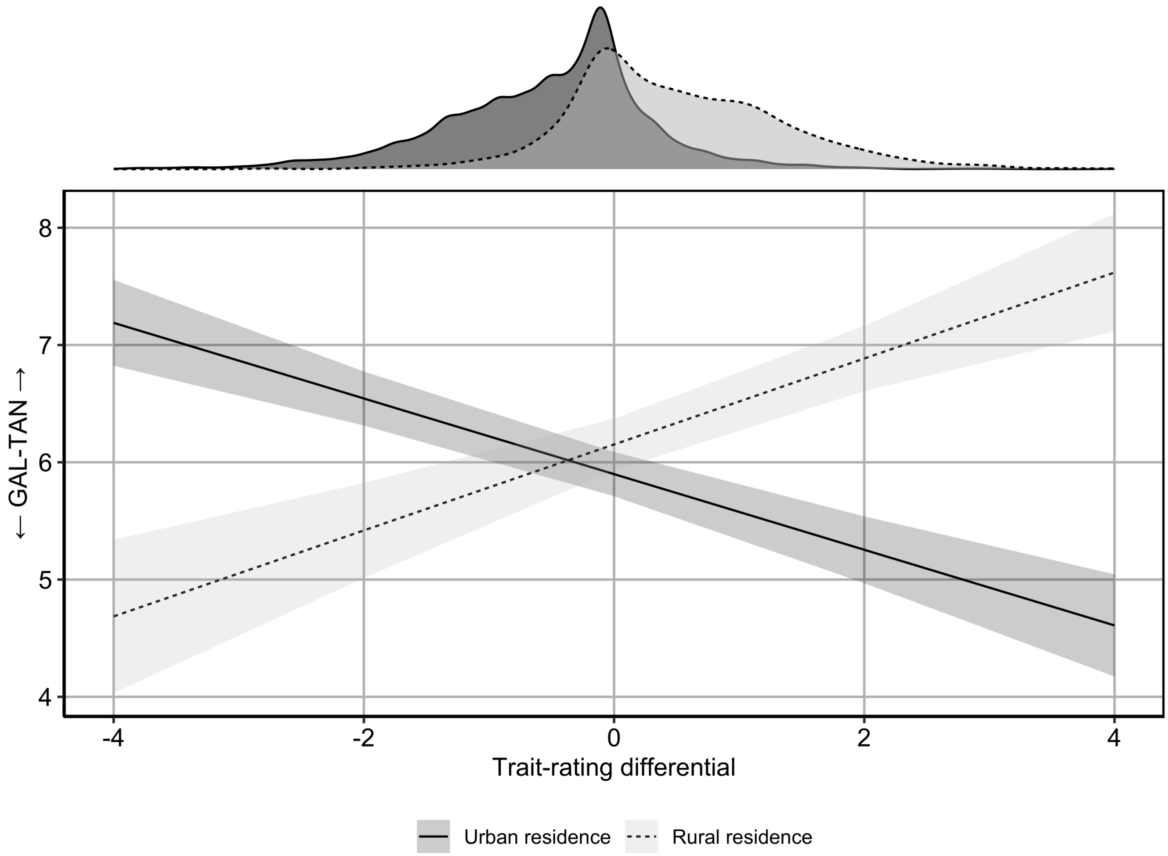

Note: Predicted values of GAL-TAN voting based on OLS regression with country fixed effects. 95% confidence intervals displayed. Models control for gender, age, education, income, migration background, and left-right self-placement. For full model results with a standardised version of the trait-rating differential, see Table A.6 in Appendix B.

Table A.6: OLS regression results: GAL-TAN voting on place-based affective polarisation, conditional on urban-rural self-classifications (trait-rating differential).

	(1)	(2)	(3)
Trait-rating differential (Std.)	−0.486***	−0.489***	−0.291***
	(0.046)	(0.049)	(0.041)
Rural residence (b.=urban residence)	0.254**	0.216*	0.168*
	(0.095)	(0.103)	(0.085)
Gender (b.=male)		−0.198**	−0.038
		(0.074)	(0.062)
Age (Std.)		0.095*	0.110***
		(0.038)	(0.031)
Education (b.=low)		−0.448***	−0.330***
		(0.083)	(0.069)
Income (Deciles)		0.003	−0.024*
		(0.013)	(0.011)
Migration background (b.=no)		−0.128	−0.066
		(0.107)	(0.089)
Left-right (Std.)			1.393***
			(0.028)
Trait-rating differential (Std.) X Rural residence	1.007***	0.943***	0.622***
	(0.084)	(0.090)	(0.075)
Constant	5.776***	5.993***	6.042***
	(0.101)	(0.137)	(0.114)
Country fixed effects	Yes	Yes	Yes
Num.Obs.	6,430	5,474	5,474
R2	0.067	0.079	0.365
R2 Adj.	0.065	0.076	0.363

+ $p < 0.1$, * $p < 0.05$, ** $p < 0.01$, *** $p < 0.001$

Figure A.4: Distributions of place-based resentment per country, by self-classified urban-rural residence.

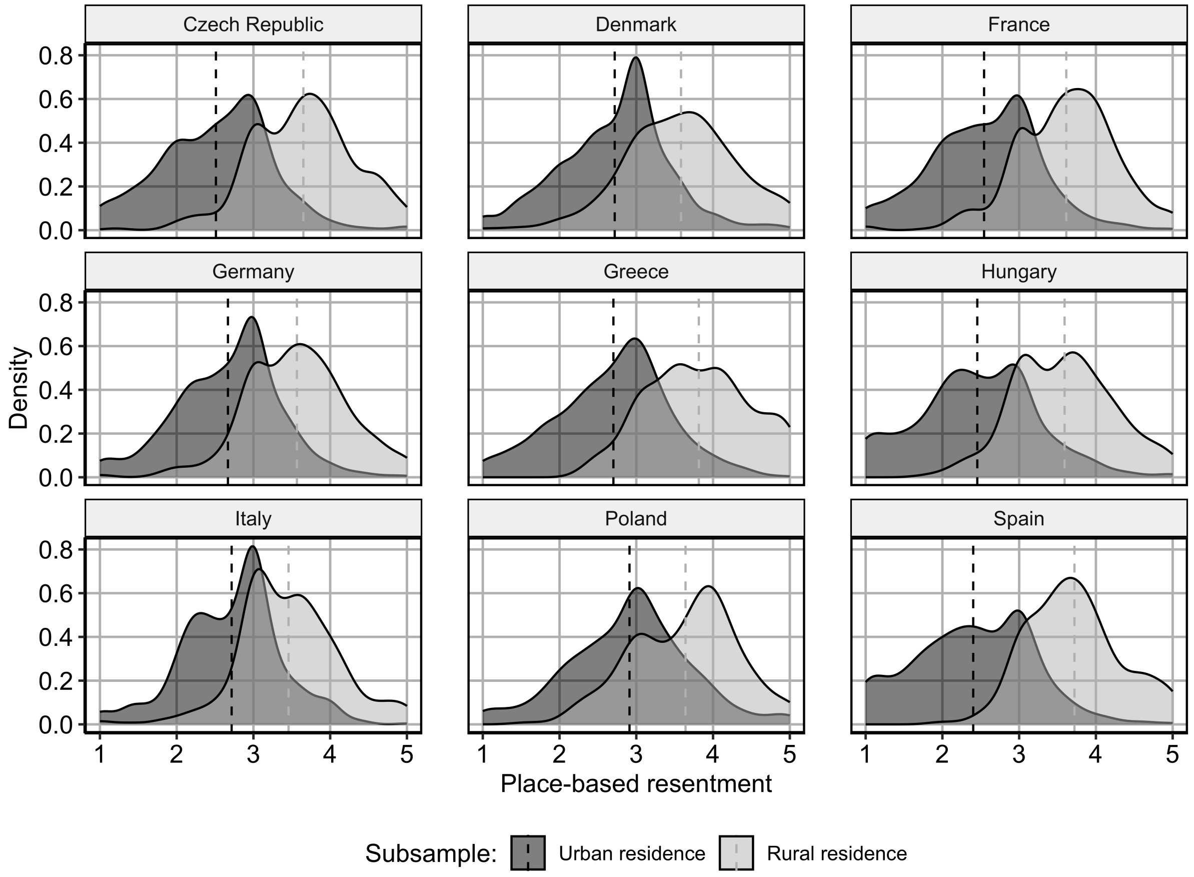

Note: Kernel density plot. The dashed lines indicate the mean values of place-based resentment for urban and rural respondents respectively.

Table A.7: Place-based resentment items.

**Economic**	Our [in] give more taxes to the state than they get back, because the money goes to [out].
**Representation (A)**	In recent years, political parties have paid too much attention to the concerns of people living in [out] and too little attention to the concerns of people living in [in].
**Representation (B)**	[Out] have too much to say in politics, while [in] are often overheard.
**Culture (A)**	People in [out] don't understand or respect the culture and lifestyle of people living in [in].
**Culture (B)**	People in [in] work harder than people in [out], because in [in] it is harder to make ends meet.

Note: Adapted from Munis (2022). If respondents see themselves as living in a very rural/rather rural place: [in] = rural areas; [out] = urban areas; If respondents see themselves as living in a very urban/rather urban place: [in] = urban areas; [out] = rural areas.

Figure A.5: Correlations between place-based resentment and place-based affective polarisation, by self-classified urban-rural residence.

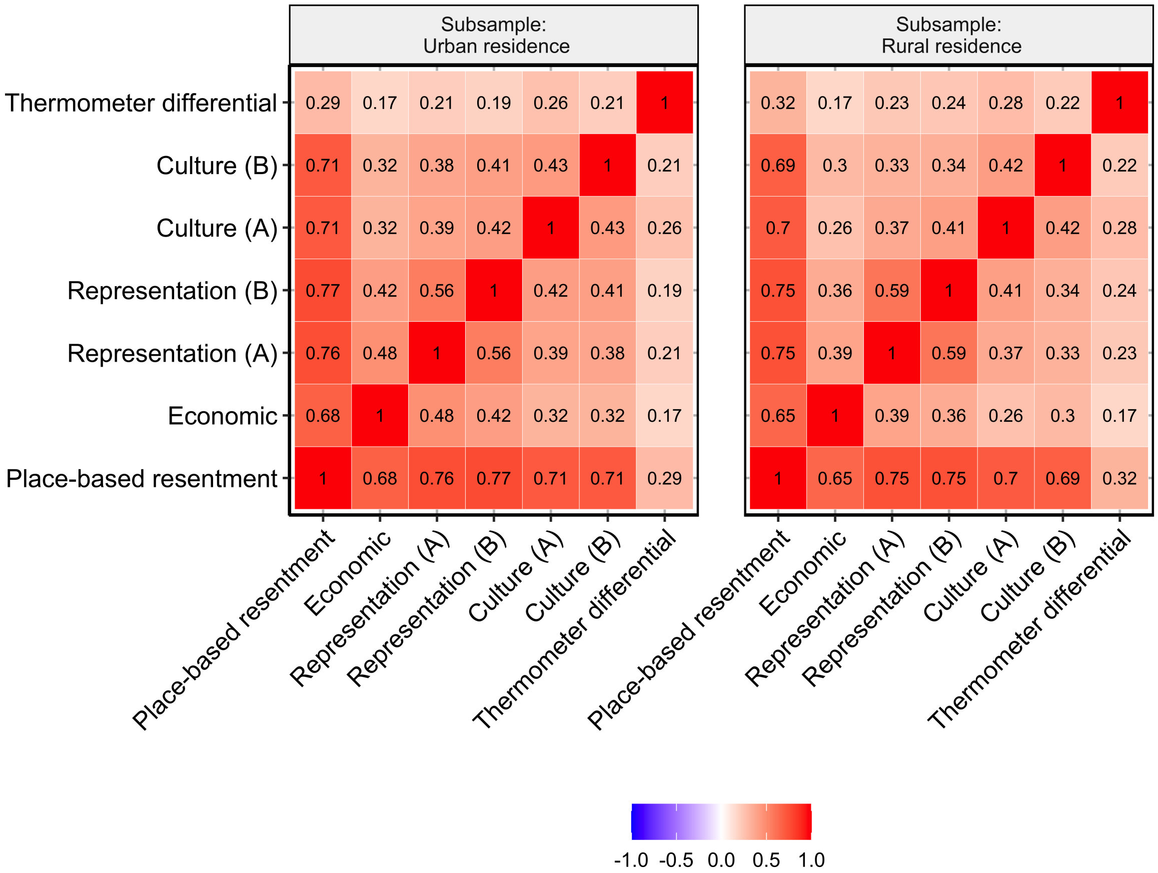

Note: Pearson's R correlation coefficients between place-based resentment and place-based affective polarisation for urban and rural respondents respectively.

Figure A.6: Distributions of place-based identity per country, by self-classified urban-rural residence.

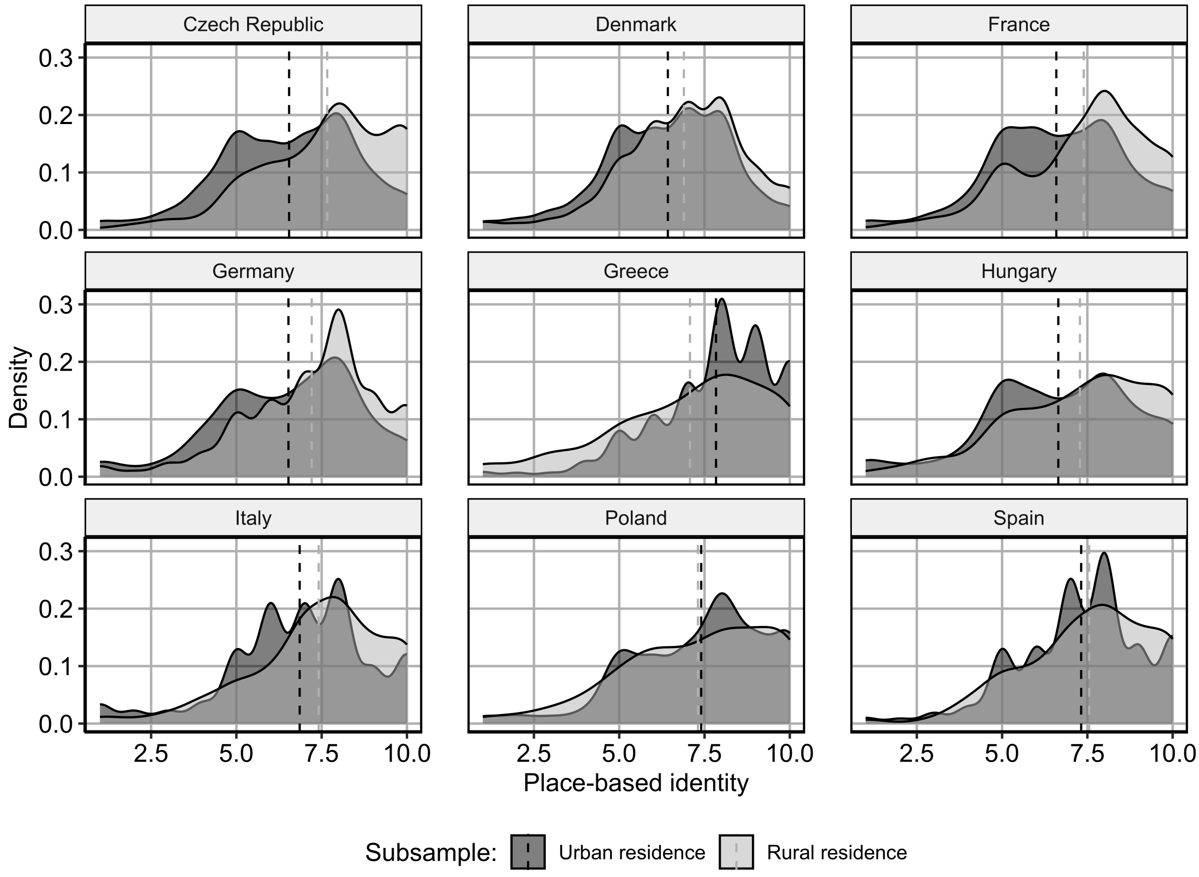

Note: Kernel density plot. Shows attachment to rural people for the rural residents, and attachment to urban people for urban residents. The dashed lines indicate the mean values of place-based identity for urban and rural respondents respectively.

Figure A.7: Distributions of attachment to place of residence per country, by self-classified urban-rural residence.

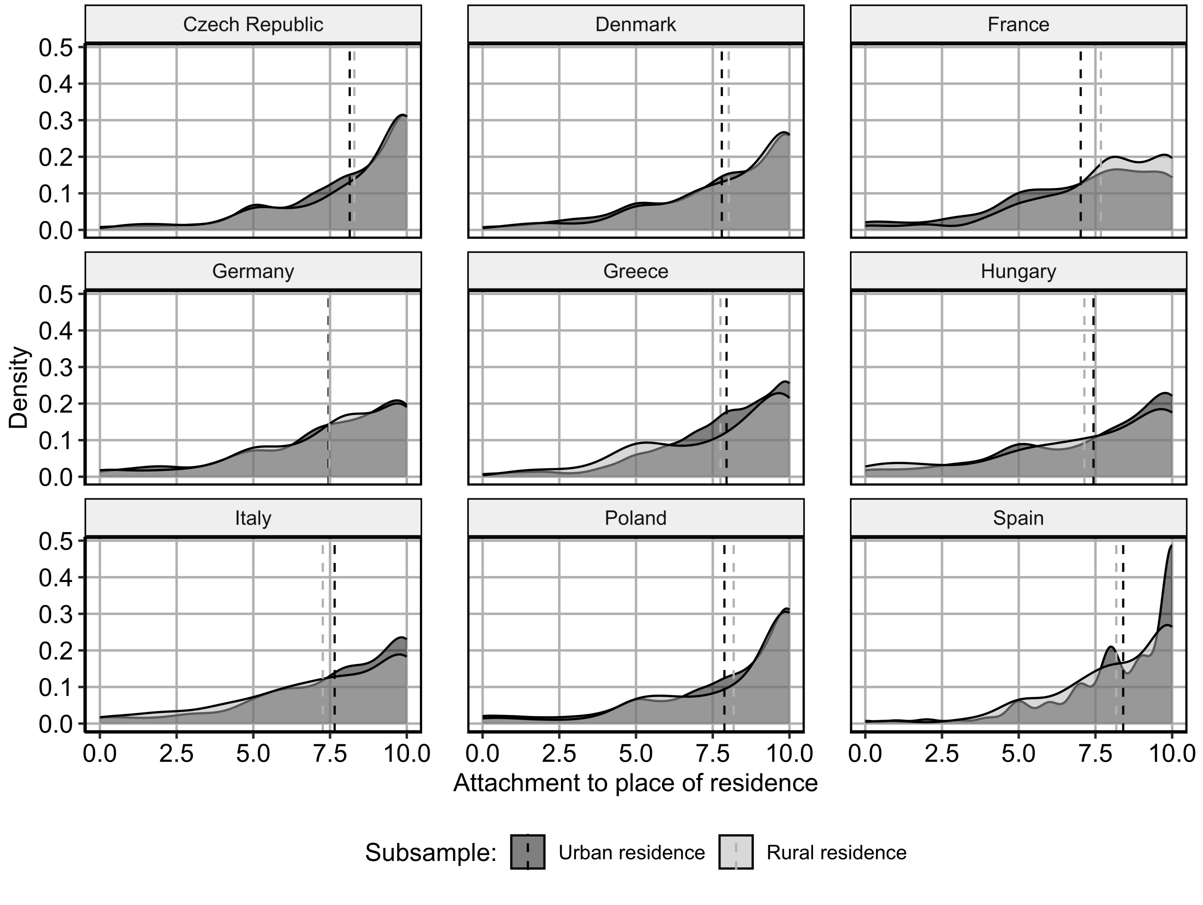

Note: Kernel density plot. The dashed lines indicate the mean values of attachment to place of residence for urban and rural respondents respectively.

Figure A.8: OLS regression results: place-based affective polarisation on place-based resentment and attachment to place of residence, by self-classified urban-rural residence.

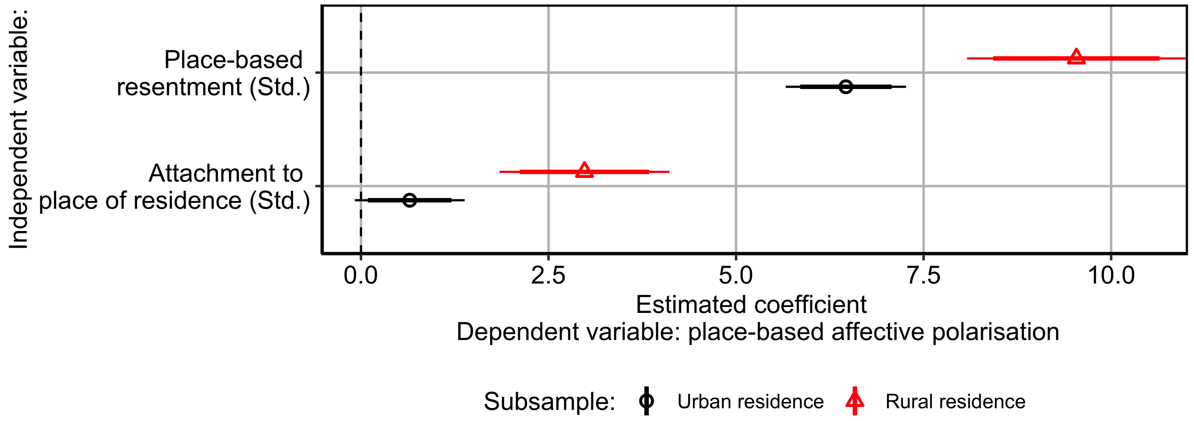

Note: OLS regression coefficients with country fixed effects. Thick and thin lines are 95% and 99% confidence intervals, respectively. Models control for gender, age, education, income, migration background, and left-right self-placement. For full model results see Table A.8 in the Appendix.

Table A.8: OLS regression results: place-based affective polarisation on place-based resentment and attachment to place of residence, by self-classified urban-rural residence.

	Urban sample			Rural sample		
	(1)	(2)	(3)	(4)	(5)	(6)
Place-based resentment (Std.)	6.335***	6.422***	6.465***	8.827***	9.535***	9.538***
	(0.282)	(0.312)	(0.311)	(0.506)	(0.567)	(0.566)
Attachment to place of residence (Std.)	0.349	0.501+	0.650*	3.244***	3.095***	2.980***
	(0.247)	(0.284)	(0.284)	(0.389)	(0.439)	(0.440)
Gender (b.=male)		0.250	0.031		−1.038	−0.975
		(0.558)	(0.558)		(0.889)	(0.887)
Age (Std.)		−0.447	−0.465		−1.647***	−1.591***
		(0.286)	(0.285)		(0.476)	(0.475)
Education (b.=low)		1.601**	1.479*		−0.319	−0.217
		(0.601)	(0.600)		(1.092)	(1.090)
Income (Deciles)		0.088	0.121		0.021	0.015
		(0.098)	(0.097)		(0.157)	(0.156)
Migration background (b.=no)		−0.023	−0.096		−3.597**	−3.585**
		(0.792)	(0.790)		(1.325)	(1.322)
Left-right (Std.)			−1.585***			1.302**
			(0.269)			(0.434)
Constant	−5.351***	−6.072***	−5.924***	10.088***	9.703***	9.839***
	(0.738)	(1.024)	(1.021)	(1.254)	(1.734)	(1.731)
Country fixed effects	Yes	Yes	Yes	Yes	Yes	Yes
Num.Obs.	6,396	5,204	5,204	2,718	2,199	2,199
R2	0.087	0.095	0.101	0.143	0.157	0.161
R2 Adj.	0.085	0.092	0.098	0.140	0.151	0.155

+ $p < 0.1$, * $p < 0.05$, ** $p < 0.01$, *** $p < 0.001$

Figure A.9: Distributions of GAL-TAN voting per country.

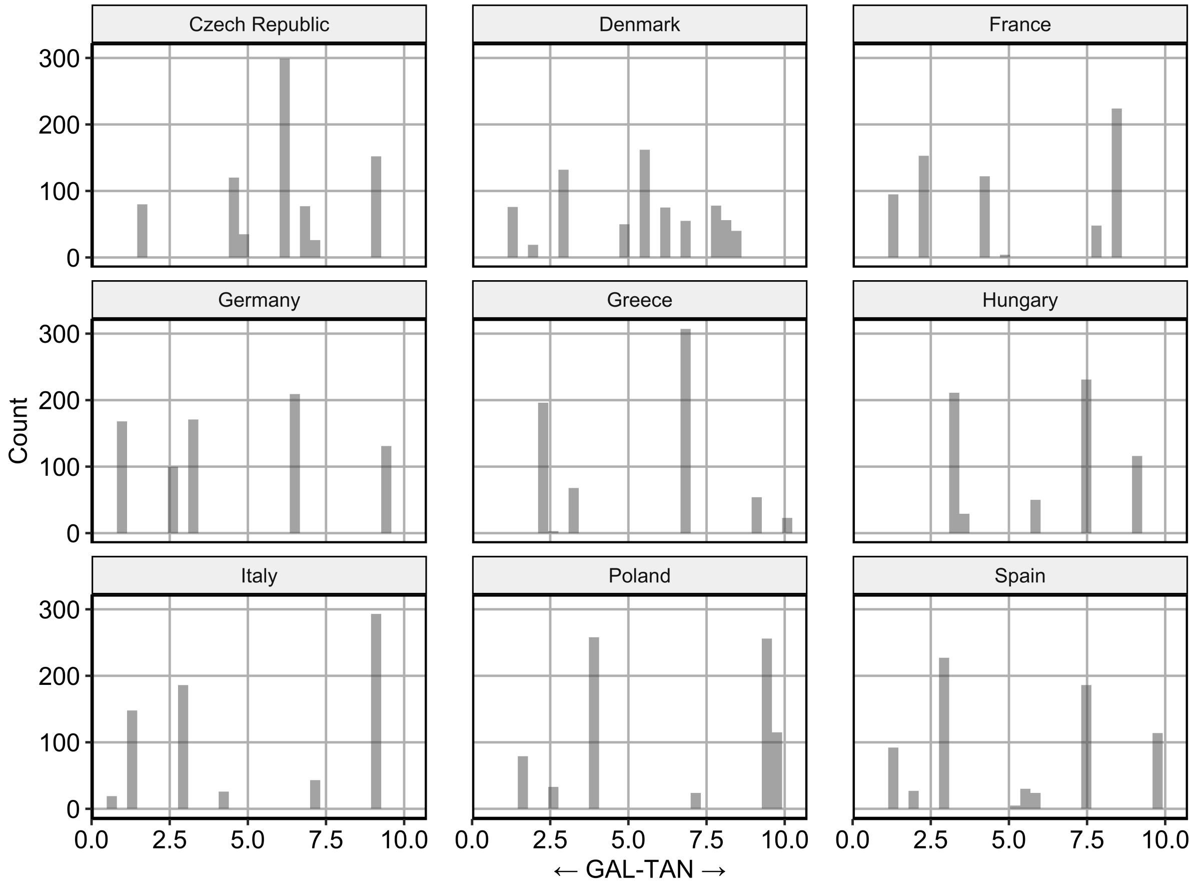

Note: Based on 2023 CHES data (Hooghe et al., 2024), using the GAL-TAN item that ranks parties from 0 ("libertarian/postmaterialist") to 10 ("traditional/authoritarian").

Figure A.10: Predicted probabilities of radical right and green voting by place-based affective polarisation, conditional on self-classified urban-rural residence.

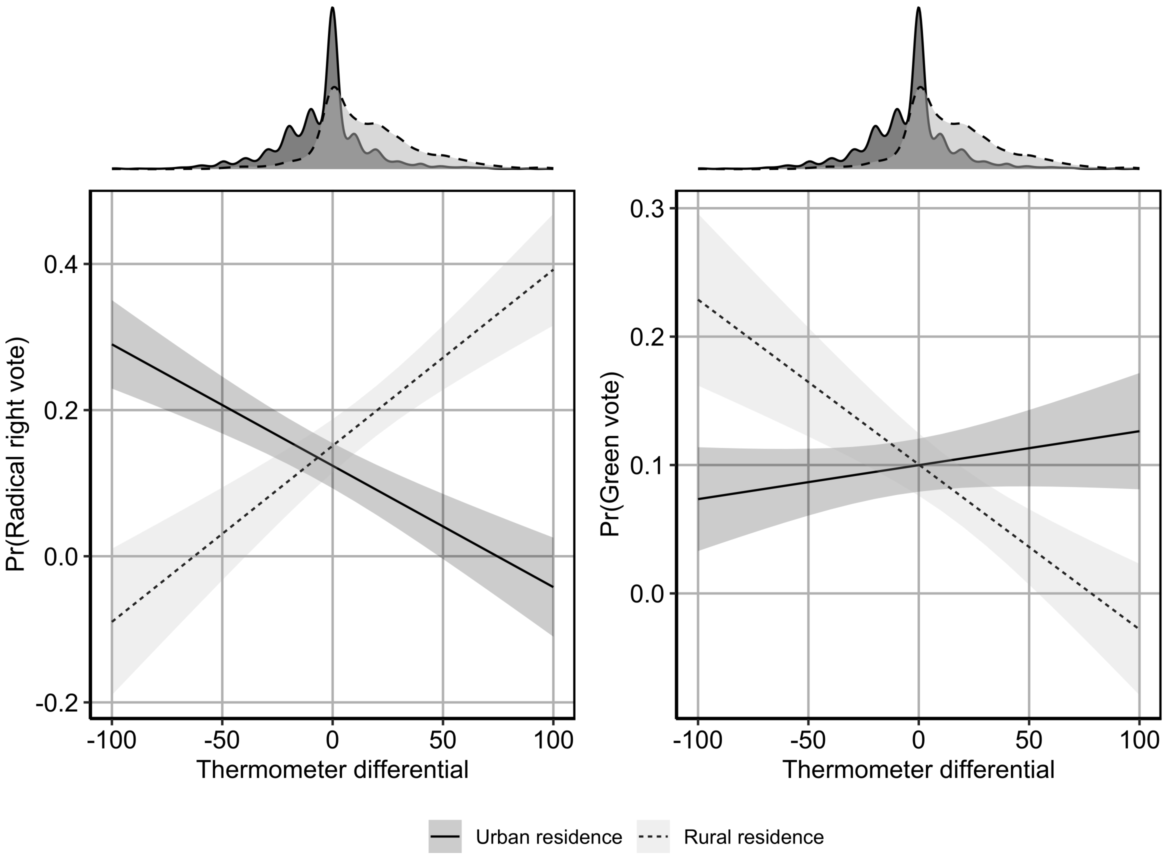

Note: Predicted probabilities of radical right and green voting based on OLS regression with country fixed effects. 95% confidence intervals displayed. Models control for gender, age, education, income, migration background, and left-right self-placement. For full model results with a standardised version of the thermometer differential, see Table A.9 in the Appendix.

Table A.9: OLS regression results: radical right and green voting on place-based affective polarisation.

	Radical right			Green		
	(1)	(2)	(3)	(4)	(5)	(6)
Thermometer differential (Std.)	−0.054***	−0.053***	−0.037***	0.010*	0.009*	0.006
	(0.007)	(0.007)	(0.006)	(0.004)	(0.004)	(0.004)
Rural residence (b.=urban residence)	0.043**	0.038**	0.031*	−0.004	−0.002	−0.001
	(0.013)	(0.014)	(0.013)	(0.008)	(0.009)	(0.009)
Gender (b.=male)		−0.034**	−0.013		0.016*	0.013+
		(0.011)	(0.010)		(0.007)	(0.007)
Age (Std.)		−0.025***	−0.023***		−0.018***	−0.019***
		(0.006)	(0.005)		(0.004)	(0.003)
Education (b.=low)		−0.076***	−0.061***		0.026***	0.023**
		(0.013)	(0.011)		(0.008)	(0.008)
Income (Deciles)		−0.003	−0.006***		0.001	0.002
		(0.002)	(0.002)		(0.001)	(0.001)
Migration background (b.=no)		−0.028+	−0.020		−0.003	−0.005
		(0.017)	(0.015)		(0.010)	(0.010)
Left-right (Std.)			0.170***			−0.029***
			(0.005)			(0.003)
Thermometer differential (Std.) X Rural residence	0.123***	0.118***	0.092***	−0.043***	−0.039***	−0.035***
	(0.012)	(0.013)	(0.011)	(0.007)	(0.008)	(0.008)
Constant	0.103***	0.154***	0.157***	0.112***	0.093***	0.093***
	(0.016)	(0.021)	(0.019)	(0.009)	(0.013)	(0.013)
Country fixed effects	Yes	Yes	Yes	Yes	Yes	Yes
Num.Obs.	6,430	5,474	5,474	6,430	5,474	5,474
R2	0.149	0.164	0.329	0.104	0.114	0.128
R2 Adj.	0.148	0.162	0.327	0.103	0.112	0.125

+ $p < 0.1$, * $p < 0.05$, ** $p < 0.01$, *** $p < 0.001$

Table A.10: Logistic regression results: radical right and green voting on place-based affective polarisation.

	Radical right			Green		
	(1)	(2)	(3)	(4)	(5)	(6)
Thermometer differential (Std.)	−0.310***	−0.308***	−0.281***	0.162*	0.133+	0.079
	(0.040)	(0.043)	(0.048)	(0.067)	(0.072)	(0.074)
Rural residence (b.=urban residence)	0.251***	0.228**	0.221*	−0.083	−0.018	−0.001
	(0.076)	(0.084)	(0.095)	(0.115)	(0.128)	(0.131)
Gender (b.=male)		−0.200**	−0.099		0.263*	0.219+
		(0.067)	(0.076)		(0.112)	(0.114)
Age (Std.)		−0.137***	−0.153***		−0.232***	−0.234***
		(0.034)	(0.039)		(0.055)	(0.056)
Education (b.=low)		−0.460***	−0.466***		0.311**	0.311*
		(0.077)	(0.088)		(0.120)	(0.122)
Income (Deciles)		−0.016	−0.041**		0.015	0.035+
		(0.012)	(0.013)		(0.019)	(0.020)
Migration background (b.=no)		−0.175+	−0.150		−0.097	−0.165
		(0.101)	(0.115)		(0.148)	(0.152)
Left-right (Std.)			1.179***			−0.583***
			(0.041)			(0.059)
Thermometer differential (Std.) X Rural residence	0.664***	0.647***	0.623***	−0.659***	−0.586***	−0.499***
	(0.067)	(0.072)	(0.082)	(0.119)	(0.128)	(0.131)
Constant	−2.051***	−1.789***	−2.077***	−2.046***	−2.369***	−2.511***
	(0.106)	(0.138)	(0.152)	(0.122)	(0.187)	(0.191)
Country fixed effects	Yes	Yes	Yes	Yes	Yes	Yes
Num.Obs.	6,430	5,474	5,474	6,430	5,474	5,474

+ $p < 0.1$, * $p < 0.05$, ** $p < 0.01$, *** $p < 0.001$

Figure A.11: OLS regression results: place-based affective polarisation on place-based resentment and place-based identity, by self-classified urban-rural residence (full variable).

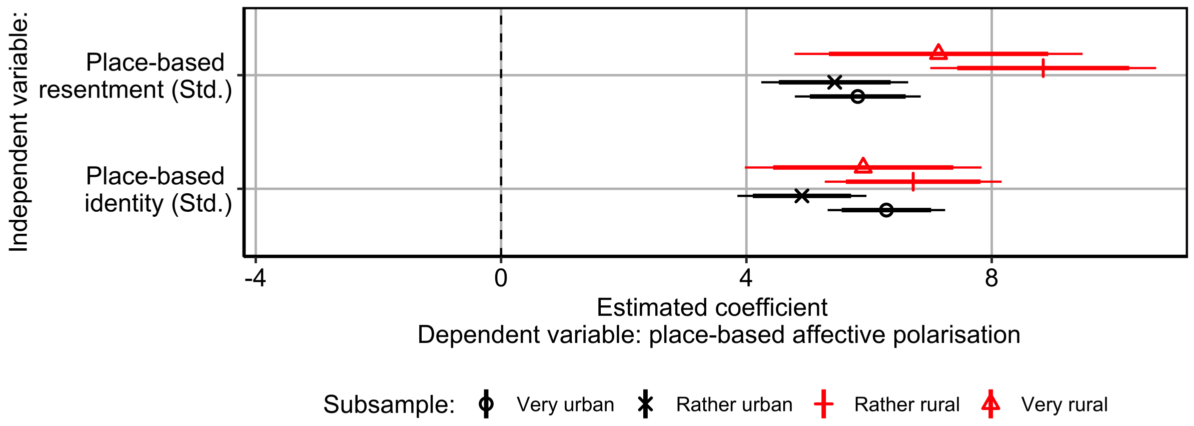

Note: OLS regression coefficients with country fixed effects. Thick and thin lines are 95% and 99% confidence intervals, respectively. Models control for gender, age, education, income, migration background, and left-right self-placement. For full model results see Table A.11 in the Appendix.

Table A.11: OLS regression results: place-based affective polarisation on place-based resentment and place-based identity, by self-classified urban-rural residence (full variable).

	Very urban sample			Rather urban sample			Rather rural sample			Very rural sample		
	(1)	(2)	(3)	(4)	(5)	(6)	(7)	(8)	(9)	(10)	(11)	(12)
Place-based resentment (Std.)	5.648***	5.795***	5.816***	5.538***	5.376***	5.440***	7.962***	8.839***	8.840***	6.612***	6.927***	7.132***
	(0.361)	(0.400)	(0.398)	(0.421)	(0.467)	(0.465)	(0.620)	(0.715)	(0.715)	(0.851)	(0.913)	(0.912)
Place-based identity (Std.)	5.734***	6.213***	6.282***	4.808***	4.787***	4.905***	6.233***	6.713***	6.719***	6.625***	6.259***	5.905***
	(0.330)	(0.373)	(0.372)	(0.363)	(0.409)	(0.409)	(0.482)	(0.556)	(0.560)	(0.676)	(0.742)	(0.749)
Gender (b.=male)		−0.159	−0.448		−0.403	−0.576		−0.436	−0.437		−3.488*	−3.244*
		(0.729)	(0.728)		(0.800)	(0.798)		(1.064)	(1.064)		(1.460)	(1.457)
Age (Std.)		−0.639+	−0.631+		−0.625	−0.624		−1.340*	−1.340*		−1.855*	−1.683*
		(0.370)	(0.368)		(0.406)	(0.405)		(0.571)	(0.572)		(0.763)	(0.762)
Education (b.=low)		1.017	0.902		1.617+	1.458+		0.989	0.986		−2.287	−2.162
		(0.774)	(0.771)		(0.878)	(0.875)		(1.264)	(1.265)		(1.914)	(1.906)
Income (Deciles)		−0.114	−0.078		0.119	0.157		−0.077	−0.077		0.175	0.175
		(0.128)	(0.127)		(0.139)	(0.139)		(0.187)	(0.188)		(0.258)	(0.257)
Migration background (b.=no)		−1.152	−1.247		0.269	0.197		−3.326*	−3.329*		−5.303*	−5.391*
		(1.010)	(1.006)		(1.167)	(1.162)		(1.580)	(1.581)		(2.195)	(2.186)
Left-right (Std.)			−1.763***			−1.742***			−0.045			1.979**
			(0.341)			(0.396)			(0.527)			(0.709)
Constant	−4.264***	−3.316*	−3.007*	−4.508***	−5.237***	−5.115***	9.941***	8.181***	8.180***	10.418***	12.240***	12.475***
	(1.035)	(1.396)	(1.391)	(0.989)	(1.425)	(1.419)	(1.679)	(2.256)	(2.257)	(1.787)	(2.574)	(2.564)
Country fixed effects	Yes	Yes	Yes	Yes	Yes	Yes	Yes	Yes	Yes	Yes	Yes	Yes
Num.Obs.	3,472	2,841	2,841	2,924	2,363	2,363	1,759	1,407	1,407	959	792	792
R2	0.160	0.178	0.185	0.129	0.131	0.138	0.201	0.222	0.222	0.200	0.216	0.223
R2 Adj.	0.158	0.173	0.181	0.126	0.126	0.132	0.197	0.214	0.213	0.192	0.200	0.207

+ $p < 0.1$, * $p < 0.05$, ** $p < 0.01$, *** $p < 0.001$

Figure A.12: Predicted values of GAL-TAN voting variable by place-based affective polarisation, conditional on urban-rural self-classifications (full variable).

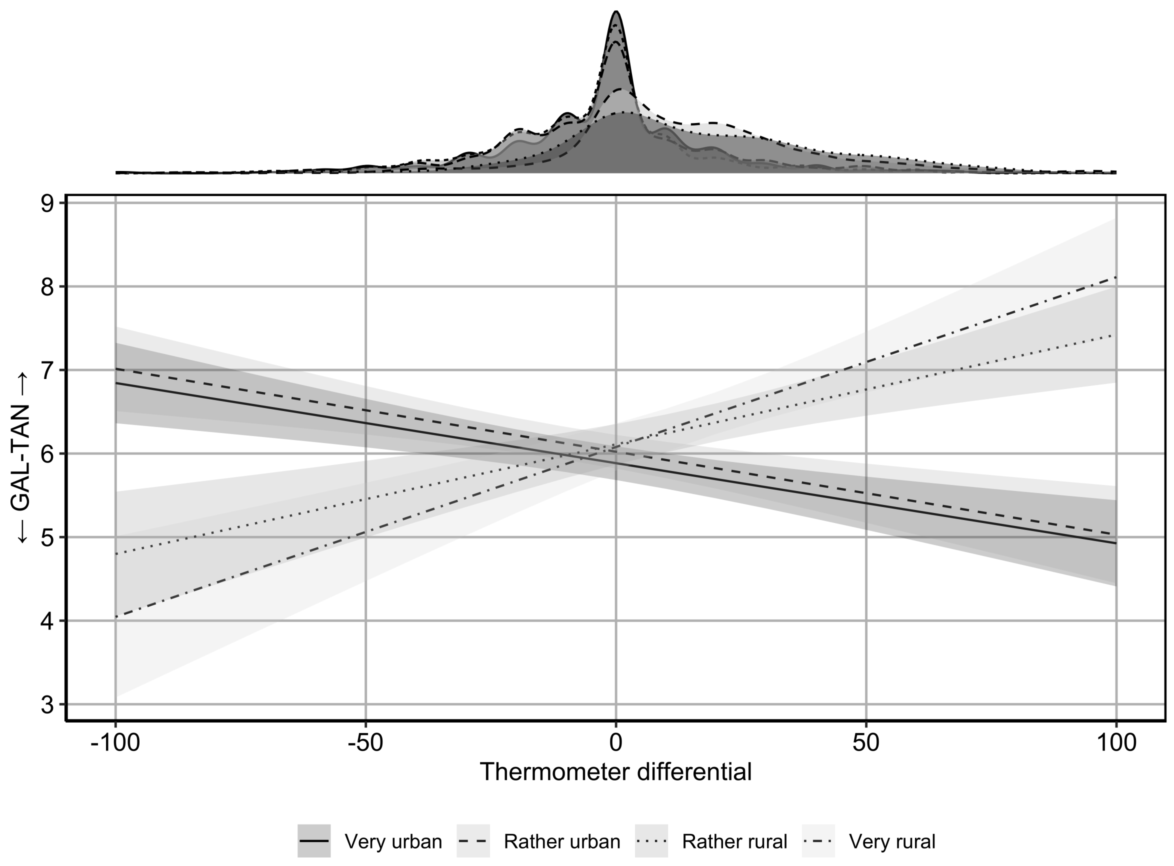

Note: Predicted values of GAL-TAN voting based on OLS regression with country fixed effects. 95% confidence intervals displayed. Models control for gender, age, education, income, migration background, and left-right self-placement. For full model results with a standardised version of the thermometer differential, see Table A.12 in the Appendix.

Table A.12: OLS regression results: GAL-TAN voting on place-based affective polarisation, conditional on self-classified urban-rural residence (full variable).

	(1)	(2)	(3)
Thermometer differential (Std.)	−0.359***	−0.348***	−0.216***
	(0.060)	(0.063)	(0.052)
Urban-rural (full variable)			
(b.=very urban residence)			
Rather urban residence	0.227**	0.214*	0.137+
	(0.087)	(0.095)	(0.078)
Rather rural residence	0.408***	0.352**	0.246*
	(0.111)	(0.121)	(0.100)
Very rural residence	0.405**	0.288+	0.221+
	(0.140)	(0.152)	(0.126)
Gender (b.=male)		−0.219**	−0.051
		(0.075)	(0.062)
Age (Std.)		0.090*	0.107***
		(0.038)	(0.032)
Education (b.=low)		−0.454***	−0.330***
		(0.084)	(0.069)
Income (Deciles)		0.005	−0.023*
		(0.013)	(0.011)
Migration background (b.=no)		−0.123	−0.059
		(0.108)	(0.089)
Left-right (Std.)			1.401***
			(0.028)
Thermometer differential (Std.) X Rather urban residence	0.027	0.016	−0.008
	(0.089)	(0.094)	(0.078)
Thermometer differential (Std.) X Rather rural residence	0.761***	0.714***	0.511***
	(0.100)	(0.107)	(0.088)
Thermometer differential (Std.) X Very rural residence	0.951***	0.933***	0.673***
	(0.120)	(0.127)	(0.106)
Constant	5.677***	5.908***	5.973***
	(0.110)	(0.146)	(0.121)
Country fixed effects	Yes	Yes	Yes
Num.Obs.	6,430	5,474	5,474
R2	0.061	0.074	0.365
R2 Adj.	0.059	0.071	0.363

+ p < 0.1, * p < 0.05, ** p < 0.01, *** p < 0.001

Figure A.13: Marginal effects of self-classified rural residence/urban residence on GAL-TAN voting, conditional on place-based affective polarisation.

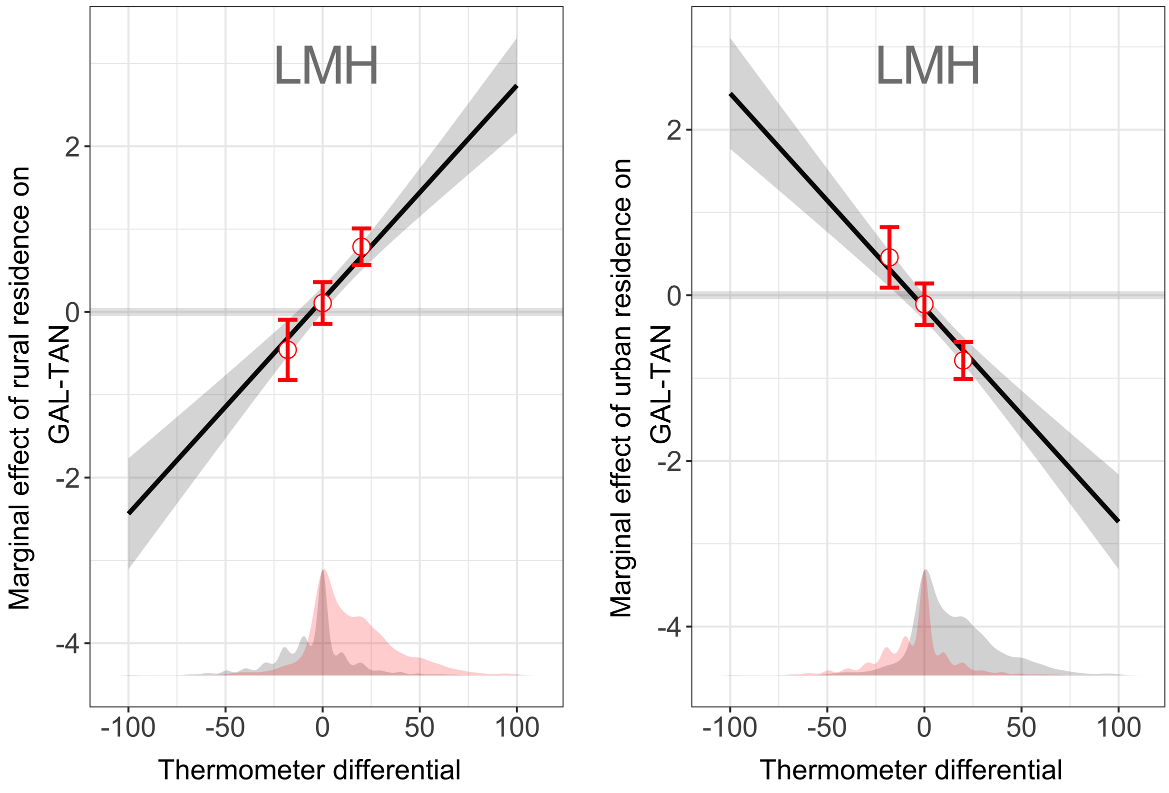

Note: Binning estimator with country fixed effects implemented using the R package `interflex` (Hainmueller et al., 2019). 95% confidence intervals displayed. Models control for gender, age, education, income, migration background, and left-right self-placement.

Figure A.14: OLS regression results: place-based affective polarisation on place-based resentment and place-based identity, by self-classified urban-rural residence (controlling for immigration attitudes).

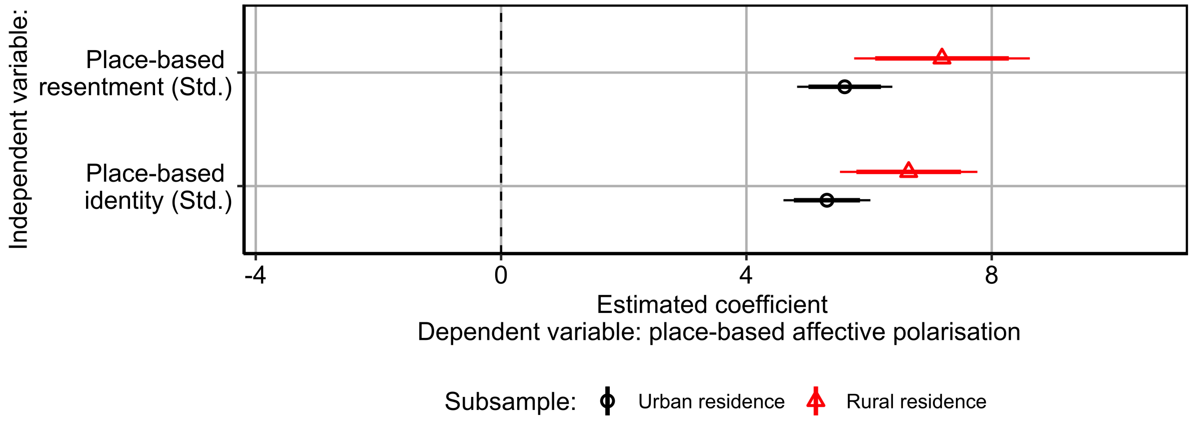

Note: OLS regression coefficients with country fixed effects. Thick and thin lines are 95% and 99% confidence intervals, respectively. Models control for gender, age, education, income, migration background, and immigration attitudes. For full model results see Table A.13 in the Appendix.

Table A.13: OLS regression results: place-based affective polarisation on place-based resentment and place-based identity, by self-classified urban-rural residence (controlling for immigration attitudes).

	Urban sample<br>(1)	Rural sample<br>(2)
Place-based resentment (Std.)	5.603***	7.189***
	(0.301)	(0.556)
Place-based identity (Std.)	5.313***	6.646***
	(0.275)	(0.435)
Gender (b.=male)	−0.123	−1.793*
	(0.535)	(0.842)
Age (Std.)	−0.573*	−1.226**
	(0.270)	(0.448)
Education (b.=low)	0.962+	0.512
	(0.577)	(1.035)
Income (Deciles)	−0.044	0.071
	(0.094)	(0.148)
Migrant background (b.=no)	−0.967	−3.216*
	(0.762)	(1.257)
Immigration attitudes (Std.)	1.996***	−4.046***
	(0.278)	(0.434)
Constant	−3.423***	8.957***
	(0.989)	(1.645)
Country fixed effects	Yes	Yes
Num.Obs.	5,204	2,199
R2	0.170	0.246
R2 Adj.	0.168	0.240

+ $p < 0.1$, * $p < 0.05$, ** $p < 0.01$, *** $p < 0.001$

Figure A.15: Predicted values of GAL-TAN voting variable by place-based affective polarisation, conditional on urban-rural self-classifications (controlling for immigration attitudes).

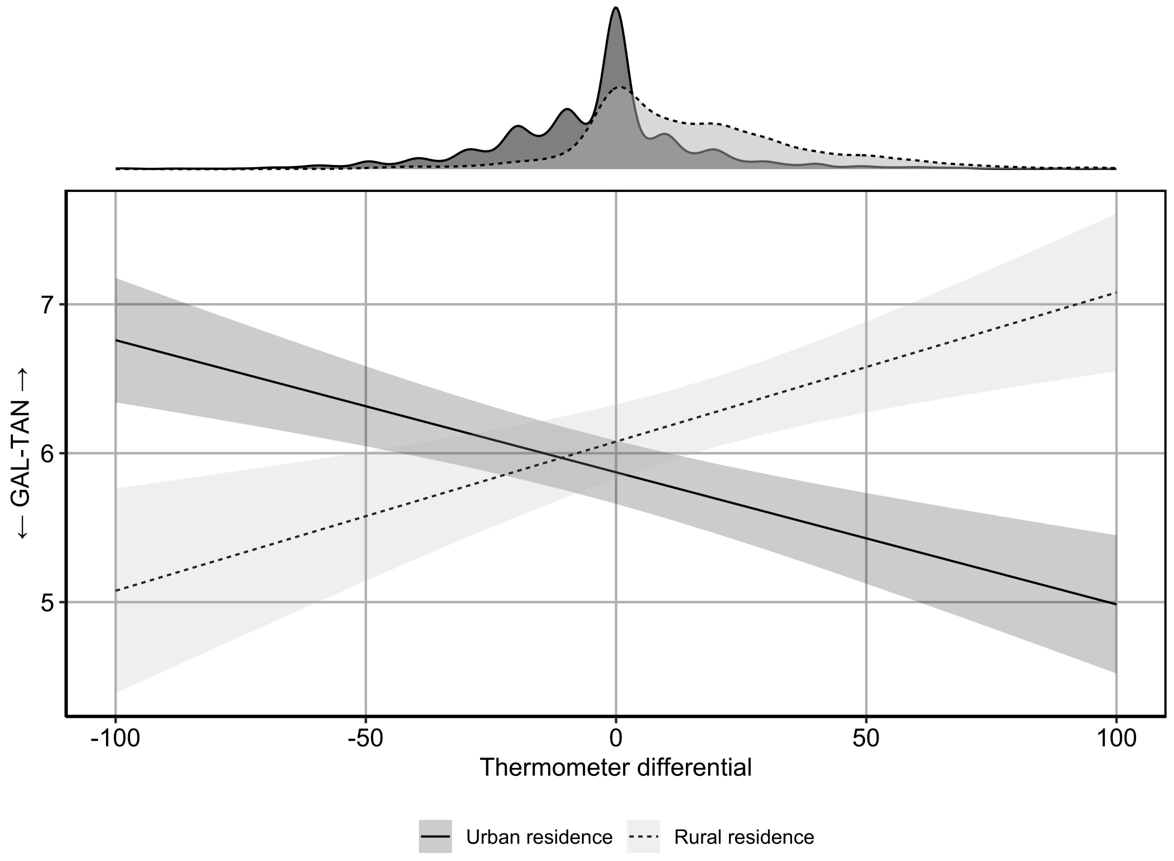

Note: Predicted values of GAL-TAN voting based on OLS regression with country fixed effects. 95% confidence intervals displayed. Models control for gender, age, education, income, migration background, and immigration attitudes. For full model results with a standardised version of the thermometer differential, see Table A.14 in the Appendix.

Table A.14: OLS regression results: GAL-TAN voting on place-based affective polarisation, conditional on self-classified urban-rural residence (controlling for immigration attitudes).

Thermometer differential (Std.)	–0.200***
	(0.044)
Rural residence (b.=urban residence)	0.223*
	(0.089)
Gender (b.=male)	–0.304***
	(0.070)
Age (Std.)	0.079*
	(0.036)
Education (b.=low)	–0.311***
	(0.079)
Income (Deciles)	0.020
	(0.012)
Migrant background (b.=no)	0.103
	(0.102)
Immigration attitudes (Std.)	–0.966***
	(0.036)
Thermometer differential (Std.) X Rural residence	0.425***
	(0.079)
Constant	5.769***
	(0.130)
Country fixed effects	Yes
Num.Obs.	5,474
R2	0.181
R2 Adj.	0.178

+ $p < 0.1$, * $p < 0.05$, ** $p < 0.01$, *** $p < 0.001$

Table A.15: Results of unpaired t-tests comparing thermometer differential scores between self-classified rural and urban residents per country (one-tailed).

Country	Estimate	t-statistic	Degrees of freedom	p-value
Czech Republic	26.722	-17.868	522.983	2.161e-56
Denmark	15.141	-11.056	461.827	1.141e-25
France	20.242	-15.828	941.872	1.624e-50
Germany	22.156	-15.439	924.071	2.577e-48
Greece	12.080	-7.440	239.046	9.034e-13
Hungary	18.757	-12.472	539.535	7.543e-32
Italy	17.455	-12.452	625.592	2.951e-32
Poland	13.269	-7.966	338.368	1.265e-14
Spain	23.652	-14.328	289.542	7.323e-36

Figure A.16: Distributions of place-based affective polarisation and affective partisan polarisation per country.

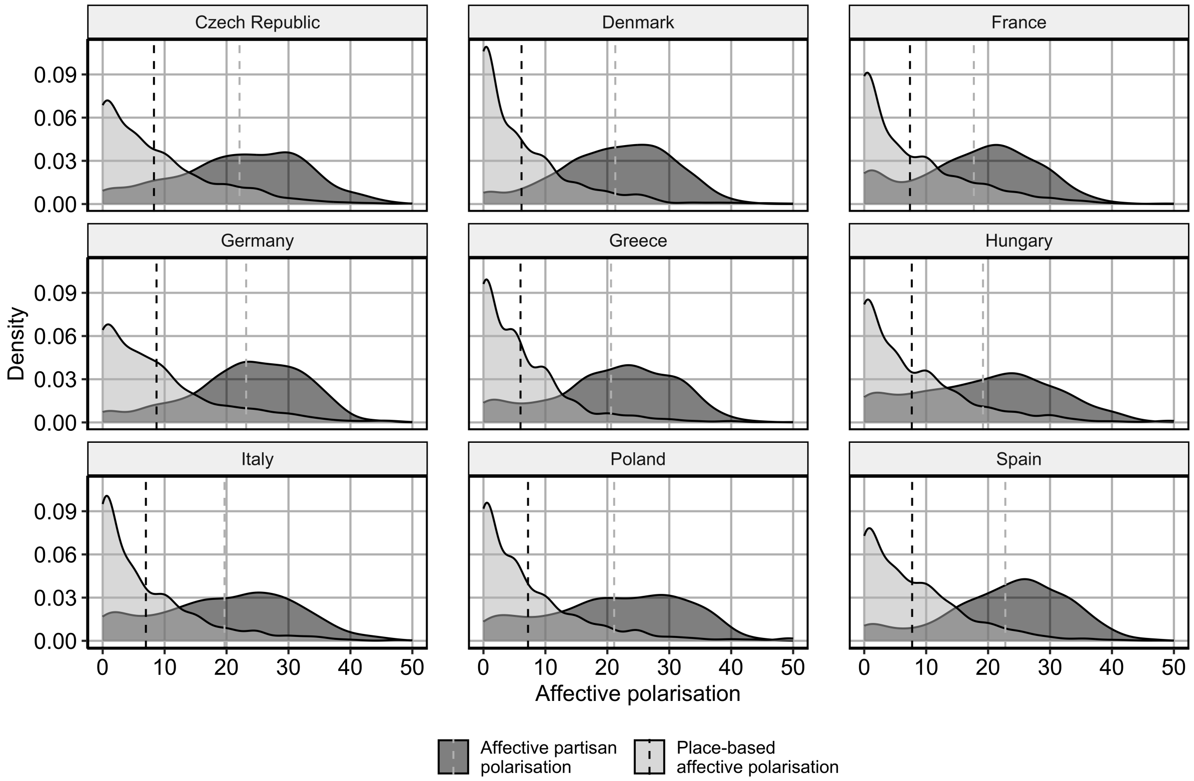

Note: Kernel density plot. Affective partisan polarisation is measured with the help of thermometer ratings asking respondents to indicate how warm or cold they feel towards different parties in their country. We rely on the unweighted spread-of-scores measure presented in Wagner (2021). Formally, we calculate the level of affective partisan polarisation as: $\text{Spread}_i = \sqrt{\frac{\sum_{p=1}^{P}(thermometer_{ip} - \overline{thermometer_i})^2}{n_p}}$, where p is the party, i is the individual respondent, and $thermometer_ip$ is the thermometer score assigned to each party p by individual i (Wagner, 2021, p.4). We use the same measure for place-based affective polarisation, calculating it as: $\text{Spread}_i = \sqrt{\frac{\sum_{g=1}^{G}(thermometer_{ig} - \overline{thermometer_i})^2}{n_g}}$, where g is the place-based group, i is the individual respondent, and $thermometer_ig$ is the thermometer score assigned to each place-based group g by individual i. The dashed lines indicate the mean values of place-based affective polarisation and affective partisan polarisation respectively. For both measures, higher values mean higher levels of affective polarisation.

Figure A.17: Urban-rural self-classification variables compared.

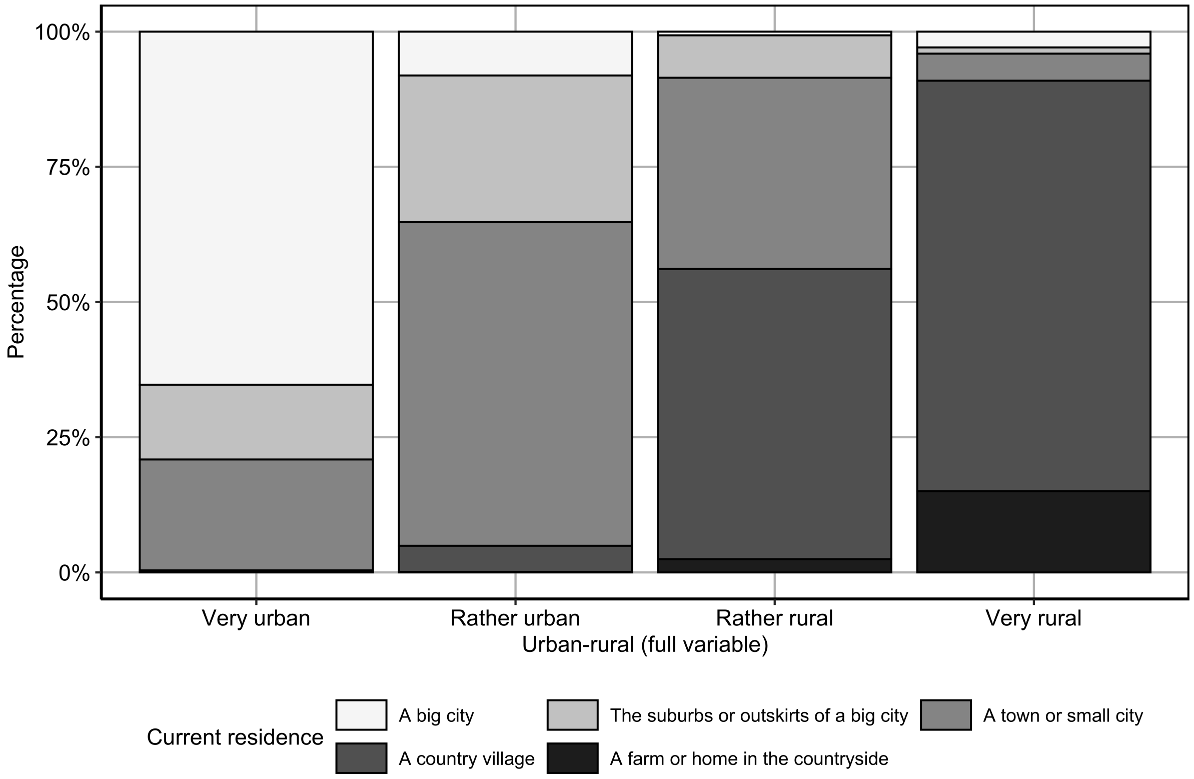

Note: Comparison of different urban-rural self-classifications. Urban-rural (full variable): "Do you live in an urban or rural area?" Answer categories are "very rural", "rather rural", "rather urban" and "very urban". Current residence: "Of the following categories, which describes best where you live?" Answer categories are "a big city", "the suburbs or outskirts of a big city", "a town or small city", "a country village" and "a farm or home in the countryside".

Table A.16: OLS regression results: place-based affective polarisation on movement indicators, by self-classified urban-rural residence.

	Urban sample (1)	Rural sample (2)
Urban move (b.=no)	−2.084***	
	(0.561)	
Rural move (b.=no)		−0.289
		(0.879)
Constant	−7.671***	18.484***
	(0.773)	(1.302)
Country fixed effects	Yes	Yes
Num.Obs.	6,396	2,718
R2	0.017	0.015
R2 Adj.	0.015	0.011

+ p < 0.1, * p < 0.05, ** p < 0.01, *** p < 0.001

Table A.17: OLS regression results: place-based affective polarisation on movement indicators, by self-classified urban-rural residence (with control variables).

	Urban sample		Rural sample	
	(1)	(2)	(3)	(4)
Urban move (b.=no)	−2.488***	−2.540***		
	(0.633)	(0.632)		
Rural move (b.=no)			−0.358	−0.375
			(0.996)	(0.994)
Gender (b.=male)	−0.047	−0.234	−0.767	−0.693
	(0.580)	(0.580)	(0.957)	(0.955)
Age (Std.)	−0.529+	−0.525+	−0.025	0.024
	(0.293)	(0.292)	(0.511)	(0.509)
Education (b.=low)	2.336***	2.229***	−1.260	−1.126
	(0.624)	(0.623)	(1.175)	(1.172)
Income (Deciles)	0.133	0.164	0.084	0.075
	(0.101)	(0.101)	(0.169)	(0.168)
Migration background (b.=no)	0.535	0.470	−4.653**	−4.623**
	(0.822)	(0.820)	(1.426)	(1.423)
Left-right (Std.)		−1.418***		1.594***
		(0.278)		(0.465)
Constant	−8.675***	−8.537***	18.239***	18.399***
	(1.068)	(1.065)	(1.828)	(1.824)
Country fixed effects	Yes	Yes	Yes	Yes
Num.Obs.	5,204	5,204	2,199	2,199
R2	0.023	0.028	0.022	0.027
R2 Adj.	0.020	0.025	0.015	0.020

+ p < 0.1, * p < 0.05, ** p < 0.01, *** p < 0.001

Table A.18: OLS regression results: place-based affective polarisation on place-based resentment and place-based identity, by self-classified urban-rural residence.

	Urban sample			Rural sample		
	(1)	(2)	(3)	(4)	(5)	(6)
Place-based resentment (Std.)	5.622***	5.630***	5.667***	7.479***	8.050***	8.075***
	(0.274)	(0.303)	(0.302)	(0.499)	(0.559)	(0.559)
Place-based identity (Std.)	5.353***	5.610***	5.691***	6.337***	6.505***	6.399***
	(0.242)	(0.273)	(0.272)	(0.392)	(0.443)	(0.447)
Gender (b.=male)		−0.292	−0.532		−1.570+	−1.526+
		(0.538)	(0.536)		(0.858)	(0.858)
Age (Std.)		−0.617*	−0.613*		−1.477**	−1.450**
		(0.272)	(0.271)		(0.455)	(0.455)
Education (b.=low)		1.235*	1.089+		−0.110	−0.056
		(0.579)	(0.576)		(1.053)	(1.053)
Income (Deciles)		−0.012	0.025		0.003	0.000
		(0.094)	(0.094)		(0.151)	(0.151)
Migration background (b.=no)		−0.477	−0.567		−4.067**	−4.051**
		(0.762)	(0.759)		(1.278)	(1.277)
Left-right (Std.)			−1.762***			0.726+
			(0.257)			(0.421)
Constant	−4.288***	−4.018***	−3.811***	10.076***	9.998***	10.069***
	(0.712)	(0.990)	(0.986)	(1.213)	(1.673)	(1.673)
Country fixed effects	Yes	Yes	Yes	Yes	Yes	Yes
Num.Obs.	6,396	5,204	5,204	2,718	2,199	2,199
R2	0.152	0.162	0.170	0.199	0.215	0.217
R2 Adj.	0.150	0.160	0.167	0.196	0.210	0.211

+ $p < 0.1$, * $p < 0.05$, ** $p < 0.01$, *** $p < 0.001$

Table A.19: OLS regression results: place-based affective polarisation on place-based resentment and place-based identity, conditional on self-classified urban-rural residence.

	(1)	(2)	(3)	(4)	(5)	(6)
Place-based resentment (Std.)	5.656***	5.654***	5.669***	6.114***	6.220***	6.224***
	(0.276)	(0.305)	(0.304)	(0.240)	(0.265)	(0.264)
Place-based identity (Std.)	5.741***	5.984***	6.063***	5.375***	5.652***	5.705***
	(0.205)	(0.232)	(0.232)	(0.244)	(0.275)	(0.274)
Rural residence (b.=urban residence)	9.651***	9.596***	9.638***	10.612***	10.774***	10.792***
	(0.595)	(0.676)	(0.675)	(0.524)	(0.598)	(0.597)
Gender (b.=male)		−0.588	−0.712		−0.560	−0.689
		(0.456)	(0.456)		(0.456)	(0.456)
Age (Std.)		−0.809***	−0.819***		−0.748**	−0.760**
		(0.234)	(0.233)		(0.233)	(0.233)
Education (b.=low)		0.909+	0.818		0.864+	0.775
		(0.509)	(0.508)		(0.509)	(0.508)
Income (Deciles)		0.001	0.019		0.001	0.019
		(0.080)	(0.080)		(0.080)	(0.080)
Migration background (b.=no)		−1.587*	−1.632*		−1.636*	−1.679*
		(0.656)	(0.655)		(0.656)	(0.655)
Left-right (Std.)			−1.071***			−1.104***
			(0.220)			(0.220)
Place-based resentment (Std.) X Rural residence	1.929***	2.378***	2.339***			
	(0.545)	(0.607)	(0.607)			
Place-based identity (Std.) X Rural residence				1.338**	1.254*	1.346**
				(0.442)	(0.501)	(0.500)
Constant	−2.803***	−2.696**	−2.639**	−2.707***	−2.532**	−2.490**
	(0.623)	(0.863)	(0.862)	(0.622)	(0.862)	(0.861)
Country fixed effects	Yes	Yes	Yes	Yes	Yes	Yes
Num.Obs.	9,114	7,403	7,403	9,114	7,403	7,403
R2	0.290	0.302	0.304	0.289	0.301	0.303
R2 Adj.	0.289	0.300	0.302	0.289	0.299	0.302

+ $p < 0.1$, * $p < 0.05$, ** $p < 0.01$, *** $p < 0.001$

Table A.20: OLS regression results: place-based affective polarisation on place-based resentment and place-based identity, by self-classified urban-rural residence (per country).

	Czech Republic		Denmark		France		Germany		Greece	
	Urban	Rural	Urban	Rural	Urban	Rural	Urban	Rural	Urban	Rural
	(1)	(2)	(3)	(4)	(5)	(6)	(7)	(8)	(9)	(10)
Place-based resentment (Std.)	5.693***	10.583***	4.502***	6.853***	5.069***	7.223***	3.872***	8.802***	3.754***	8.321***
	(0.816)	(1.546)	(0.785)	(1.371)	(0.888)	(1.231)	(1.050)	(1.295)	(0.691)	(1.689)
Place-based identity (Std.)	7.921***	4.234***	3.525***	3.643**	6.483***	7.029***	7.762***	8.199***	4.439***	5.604***
	(0.706)	(1.269)	(0.709)	(1.284)	(0.762)	(0.984)	(0.804)	(1.028)	(0.691)	(1.158)
Constant	−3.605***	8.025***	−0.310	7.616***	−1.759*	7.685***	−1.489+	10.192***	−3.184***	0.821
	(0.799)	(1.707)	(0.707)	(1.565)	(0.833)	(1.317)	(0.899)	(1.375)	(0.678)	(2.212)
Num.Obs.	715	296	734	276	543	472	561	452	836	177
R2	0.218	0.211	0.078	0.141	0.187	0.202	0.175	0.236	0.088	0.242
R2 Adj.	0.216	0.205	0.075	0.135	0.184	0.199	0.172	0.233	0.086	0.233

	Hungary		Italy		Poland		Spain	
	Urban	Rural	Urban	Rural	Urban	Rural	Urban	Rural
	(11)	(12)	(13)	(14)	(15)	(16)	(17)	(18)
Place-based resentment (Std.)	7.548***	4.620**	4.600***	6.801***	8.586***	6.470***	5.028***	8.104***
	(0.813)	(1.544)	(0.940)	(1.422)	(0.781)	(1.769)	(0.730)	(1.971)
Place-based identity (Std.)	4.919***	6.799***	4.812***	8.049***	4.939***	4.385**	3.595***	5.831***
	(0.688)	(1.113)	(0.694)	(1.102)	(0.725)	(1.319)	(0.738)	(1.508)
Constant	−1.487+	7.748***	−3.761***	6.412***	−1.844*	6.142**	−4.972***	7.023**
	(0.871)	(1.677)	(0.744)	(1.383)	(0.722)	(2.009)	(0.805)	(2.302)
Num.Obs.	727	287	663	349	806	208	811	201
R2	0.174	0.158	0.104	0.207	0.197	0.137	0.090	0.159
R2 Adj.	0.172	0.152	0.101	0.203	0.195	0.128	0.088	0.150

+ $p < 0.1$, * $p < 0.05$, ** $p < 0.01$, *** $p < 0.001$

Table A.21: OLS regression results: place-based affective polarisation on place-based resentment and place-based identity, conditional on self-classified urban-rural residence (per country).

	Czech Republic		Denmark		France		Germany		Greece	
	(1)	(2)	(3)	(4)	(5)	(6)	(7)	(8)	(9)	(10)
Place-based resentment (Std.)	6.911***		4.757***		6.303***		5.234***		4.279***	
	(0.877)		(0.806)		(0.987)		(1.145)		(0.711)	
Place-based identity (Std.)		8.575***		3.791***		7.193***		8.160***		4.883***
		(0.747)		(0.737)		(0.826)		(0.840)		(0.707)
Rural residence (b.=urban residence)	12.990***	22.556***	7.800***	14.405***	9.503***	17.163***	12.257***	19.320***	1.013	13.898***
	(1.934)	(1.414)	(1.637)	(1.317)	(1.599)	(1.200)	(1.727)	(1.327)	(2.310)	(1.479)
Place-based resentment (Std.) X Rural residence	5.187**		3.207*		3.416*		5.731**		5.183**	
	(1.739)		(1.521)		(1.531)		(1.748)		(1.833)	
Place-based identity (Std.) X Rural residence		−1.789		1.687		1.474		1.502		1.517
		(1.424)		(1.428)		(1.232)		(1.310)		(1.351)
Constant	−5.020***	−6.178***	−1.348+	−1.293+	−2.710**	−3.844***	−3.121**	−2.534**	−1.359*	−4.316***
	(0.856)	(0.757)	(0.694)	(0.714)	(0.931)	(0.824)	(0.972)	(0.900)	(0.638)	(0.665)
Num.Obs.	1,011	1,011	1,010	1,010	1,015	1,015	1,013	1,013	1,013	1,013
R2	0.333	0.354	0.175	0.154	0.280	0.314	0.260	0.320	0.119	0.128
R2 Adj.	0.331	0.352	0.173	0.152	0.277	0.312	0.258	0.318	0.116	0.125

	Hungary		Italy		Poland		Spain	
	(11)	(12)	(13)	(14)	(15)	(16)	(17)	(18)
Place-based resentment (Std.)	8.148***		4.986***		9.461***		5.439***	
	(0.837)		(1.015)		(0.788)		(0.745)	
Place-based identity (Std.)		5.580***		5.014***		6.248***		4.183***
		(0.711)		(0.731)		(0.757)		(0.763)
Rural residence (b.=urban residence)	9.493***	16.834***	10.847***	15.408***	6.436**	13.641***	11.641***	22.556***
	(1.985)	(1.468)	(1.592)	(1.284)	(2.212)	(1.675)	(2.397)	(1.577)
Place-based resentment (Std.) X Rural residence	−2.080		3.565*		−1.527		3.783+	
	(1.817)		(1.725)		(1.958)		(2.051)	
Place-based identity (Std.) X Rural residence		1.731		3.923**		−0.662		2.557
		(1.366)		(1.282)		(1.578)		(1.665)
Constant	−2.154*	−5.488***	−4.154***	−4.846***	−1.007	−2.149**	−4.268***	−8.114***
	(0.898)	(0.788)	(0.803)	(0.749)	(0.727)	(0.763)	(0.814)	(0.690)
Num.Obs.	1,014	1,014	1,012	1,012	1,014	1,014	1,012	1,012
R2	0.215	0.209	0.193	0.234	0.187	0.128	0.241	0.223
R2 Adj.	0.212	0.207	0.191	0.232	0.184	0.125	0.239	0.220

+ $p < 0.1$, * $p < 0.05$, ** $p < 0.01$, *** $p < 0.001$

Table A.22: OLS regression results: in-group affect and out-group affect on place-based resentment and place-based identity, by self-classified urban-rural residence.

	Urban sample						Rural sample					
	In-group affect			Out-group affect			In-group affect			Out-group affect		
	(1)	(2)	(3)	(4)	(5)	(6)	(7)	(8)	(9)	(10)	(11)	(12)
Place-based resentment (Std.)	1.831***	1.867***	1.867***	−3.790***	−3.763***	−3.800***	3.465***	3.718***	3.727***	−4.013***	−4.332***	−4.348***
	(0.216)	(0.236)	(0.236)	(0.235)	(0.259)	(0.257)	(0.371)	(0.410)	(0.410)	(0.422)	(0.472)	(0.472)
Place-based identity (Std.)	6.575***	6.737***	6.736***	1.222***	1.126***	1.046***	7.524***	7.415***	7.378***	1.187***	0.909*	0.979**
	(0.191)	(0.213)	(0.213)	(0.208)	(0.233)	(0.232)	(0.291)	(0.325)	(0.328)	(0.331)	(0.374)	(0.378)
Gender (b.=male)		0.442	0.443		0.733	0.975*		1.629**	1.644**		3.199***	3.170***
		(0.419)	(0.420)		(0.459)	(0.457)		(0.630)	(0.630)		(0.725)	(0.725)
Age (Std.)		−0.360+	−0.360+		0.256	0.253		−0.549	−0.540		0.928*	0.910*
		(0.212)	(0.212)		(0.232)	(0.231)		(0.334)	(0.334)		(0.385)	(0.385)
Education (b.=low)		0.378	0.378		−0.858+	−0.711		0.459	0.478		0.570	0.534
		(0.451)	(0.452)		(0.494)	(0.491)		(0.773)	(0.773)		(0.889)	(0.890)
Income (Deciles)		0.182*	0.182*		0.194*	0.156+		0.078	0.077		0.075	0.077
		(0.073)	(0.073)		(0.080)	(0.080)		(0.111)	(0.111)		(0.128)	(0.128)
Migration background (b.=no)		−0.123	−0.123		0.354	0.444		−1.203	−1.198		2.863**	2.853**
		(0.595)	(0.595)		(0.651)	(0.647)		(0.937)	(0.937)		(1.079)	(1.079)
Left-right (Std.)			0.008			1.770***			0.249			−0.478
			(0.202)			(0.219)			(0.309)			(0.356)
Constant	58.422***	57.735***	57.734***	62.710***	61.753***	61.545***	67.450***	65.991***	66.015***	57.373***	55.993***	55.946***
	(0.562)	(0.772)	(0.773)	(0.612)	(0.845)	(0.840)	(0.900)	(1.227)	(1.228)	(1.026)	(1.413)	(1.413)
Country fixed effects	Yes	Yes	Yes	Yes	Yes	Yes	Yes	Yes	Yes	Yes	Yes	Yes
Num.Obs.	6,396	5,204	5,204	6,396	5,204	5,204	2,718	2,199	2,199	2,718	2,199	2,199
R2	0.195	0.207	0.207	0.056	0.058	0.069	0.268	0.272	0.272	0.051	0.070	0.071
R2 Adj.	0.193	0.204	0.204	0.055	0.055	0.067	0.265	0.267	0.267	0.047	0.064	0.064

+ $p < 0.1$, * $p < 0.05$, ** $p < 0.01$, *** $p < 0.001$

Table A.23: OLS regression results: GAL-TAN voting on place-based affective polarisation, conditional on self-classified urban-rural residence.

	(1)	(2)	(3)
Thermometer differential (Std.)	−0.357***	−0.350***	−0.226***
	(0.044)	(0.047)	(0.039)
Rural residence (b.=urban residence)	0.301***	0.229*	0.172*
	(0.087)	(0.094)	(0.078)
Gender (b.=male)		−0.220**	−0.051
		(0.075)	(0.062)
Age (Std.)		0.085*	0.104**
		(0.038)	(0.032)
Education (b.=low)		−0.462***	−0.335***
		(0.084)	(0.069)
Income (Deciles)		0.005	−0.023*
		(0.013)	(0.011)
Migration background (b.=no)		−0.129	−0.063
		(0.108)	(0.089)
Left-right (Std.)			1.403***
			(0.028)
Thermometer differential (Std.) X Rural residence	0.829***	0.798***	0.582***
	(0.078)	(0.083)	(0.069)
Constant	5.794***	6.022***	6.048***
	(0.101)	(0.138)	(0.114)
Country fixed effects	Yes	Yes	Yes
Num.Obs.	6,430	5,474	5,474
R2	0.060	0.073	0.364
R2 Adj.	0.058	0.070	0.362

+ $p < 0.1$, * $p < 0.05$, ** $p < 0.01$, *** $p < 0.001$

Table A.24: OLS regression results: GAL-TAN voting on place-based affective polarisation, conditional on self-classified urban-rural residence (including interaction with place-based resentment).

	(1)	(2)	(3)
Thermometer differential (Std.)	−0.353***	−0.350***	−0.200***
	(0.046)	(0.049)	(0.041)
Place-based resentment (Std.)	−0.014	0.001	−0.090*
	(0.048)	(0.051)	(0.042)
Rural residence (b.=urban residence)	0.175+	0.120	0.065
	(0.104)	(0.113)	(0.094)
Gender (b.=male)		−0.222**	−0.060
		(0.075)	(0.062)
Age (Std.)		0.079*	0.094**
		(0.038)	(0.032)
Education (b.=low)		−0.461***	−0.326***
		(0.084)	(0.069)
Income (Deciles)		0.005	−0.022*
		(0.013)	(0.011)
Migration background (b.=no)		−0.130	−0.058
		(0.108)	(0.089)
Left-right (Std.)			1.406***
			(0.028)
Thermometer differential (Std.) X Rural residence	0.775***	0.757***	0.506***
	(0.082)	(0.087)	(0.072)
Place-based resentment (Std.) X Rural residence	0.209*	0.160	0.288***
	(0.096)	(0.104)	(0.086)
Constant	5.790***	6.022***	6.014***
	(0.102)	(0.139)	(0.115)
Country fixed effects	Yes	Yes	Yes
Num.Obs.	6,430	5,474	5,474
R2	0.061	0.073	0.366
R2 Adj.	0.059	0.070	0.363

+ $p < 0.1$, * $p < 0.05$, ** $p < 0.01$, *** $p < 0.001$

Table A.25: OLS regression results: GAL-TAN voting on place-based affective polarisation, conditional on self-classified urban-rural residence (per country).

	Czech Republic (1)	Denmark (2)	France (3)	Germany (4)	Greece (5)
Thermometer differential (Std.)	–0.233*	–0.496***	–0.439*	–0.495***	–0.283*
	(0.092)	(0.113)	(0.186)	(0.137)	(0.137)
Rural residence (b.=urban residence)	–0.028	0.339+	0.707**	0.351	–0.137
	(0.202)	(0.194)	(0.258)	(0.234)	(0.283)
Thermometer differential (Std.) X Rural residence	0.545**	1.219***	1.044***	1.193***	0.684*
	(0.171)	(0.197)	(0.250)	(0.198)	(0.305)
Constant	5.958***	5.021***	4.488***	4.182***	5.302***
	(0.096)	(0.093)	(0.166)	(0.137)	(0.111)
Num.Obs.	789	743	646	779	651
R2	0.015	0.067	0.055	0.063	0.010
R2 Adj.	0.011	0.063	0.051	0.059	0.005

	Hungary (6)	Italy (7)	Poland (8)	Spain (8)
Thermometer differential (Std.)	–0.373***	–0.343+	–0.335**	–0.242+
	(0.106)	(0.179)	(0.120)	(0.146)
Rural residence (b.=urban residence)	0.249	0.426	0.665*	0.129
	(0.225)	(0.302)	(0.320)	(0.348)
Thermometer differential (Std.) X Rural residence	0.910***	0.717*	0.624*	–0.096
	(0.201)	(0.282)	(0.296)	(0.311)
Constant	5.760***	5.080***	6.213***	5.201***
	(0.110)	(0.162)	(0.125)	(0.136)
Num.Obs.	637	715	765	705
R2	0.041	0.015	0.022	0.007
R2 Adj.	0.037	0.011	0.018	0.002

+ $p < 0.1$, * $p < 0.05$, ** $p < 0.01$, *** $p < 0.001$

Table A.26: OLS regression results: GAL-TAN voting on place-based affective polarisation, conditional on self-classified urban-rural residence (CHES 2019 data; per country).

	Czech Republic (1)	Denmark (2)	France (3)	Germany (4)	Greece (5)
Thermometer differential (Std.)	–0.222*	–0.545***	–0.381*	–0.478***	–0.255+
	(0.099)	(0.124)	(0.164)	(0.137)	(0.148)
Rural residence (b.=urban residence)	–0.094	0.348	0.639**	0.340	–0.090
	(0.216)	(0.218)	(0.229)	(0.234)	(0.304)
Thermometer differential (Std.) X Rural residence	0.519**	1.291***	0.933***	1.169***	0.642+
	(0.183)	(0.230)	(0.221)	(0.197)	(0.327)
Constant	5.737***	4.276***	4.386***	4.301***	5.581***
	(0.103)	(0.103)	(0.147)	(0.137)	(0.119)
Num.Obs.	789	615	642	779	651
R2	0.011	0.066	0.058	0.061	0.007
R2 Adj.	0.007	0.062	0.054	0.057	0.003

	Hungary (6)	Italy (7)	Poland (8)	Spain (8)
Thermometer differential (Std.)	–0.538**	–0.271	–0.267**	–0.248+
	(0.171)	(0.172)	(0.096)	(0.147)
Rural residence (b.=urban residence)	–0.087	0.299	0.500+	0.113
	(0.382)	(0.291)	(0.257)	(0.348)
Thermometer differential (Std.) X Rural residence	1.786***	0.644*	0.537*	–0.071
	(0.342)	(0.275)	(0.238)	(0.312)
Constant	5.547***	5.799***	6.633***	5.319***
	(0.179)	(0.156)	(0.101)	(0.137)
Num.Obs.	521	670	765	705
R2	0.054	0.012	0.022	0.007
R2 Adj.	0.049	0.008	0.018	0.002

+ p < 0.1, * p < 0.05, ** p < 0.01, *** p < 0.001

Figure A.18: Urban-rural self-classification and population density.

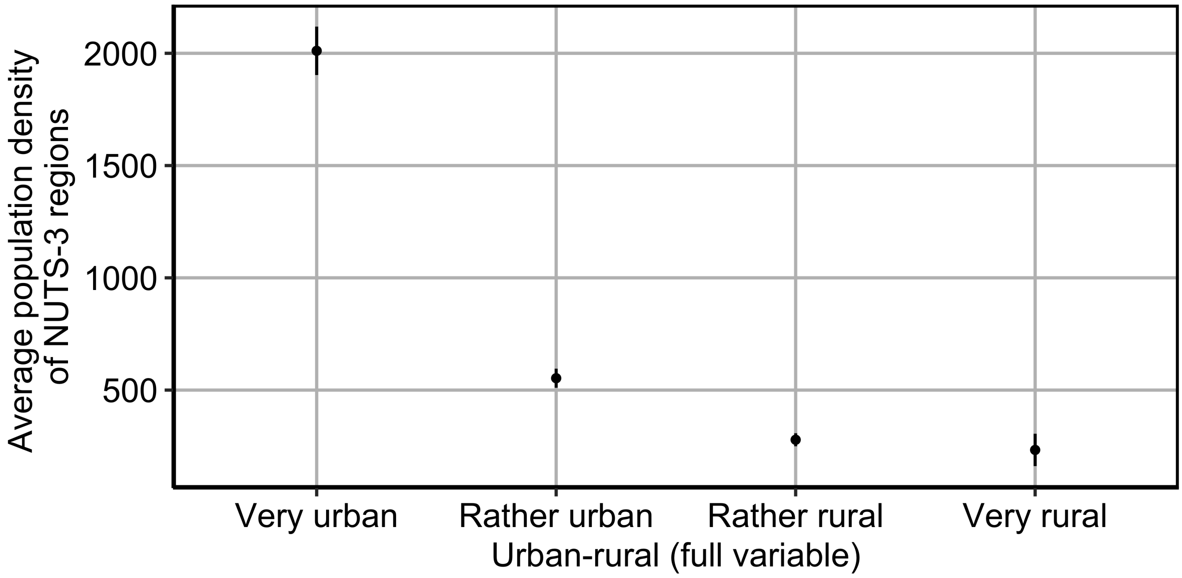

Note: Average population density of NUTS-3 regions by self-classified urban-rural residence with 95% confidence intervals. Data on population density stem from Eurostat.

Table A.27: OLS regression results: GAL-TAN voting on in-group affect and out-group affect, conditional on self-classified urban-rural residence.

	(1)	(2)	(3)
In-group affect (Std.)	−0.231***	−0.219***	−0.216***
	(0.043)	(0.046)	(0.038)
Out-group affect (Std.)	0.312***	0.314***	0.129***
	(0.043)	(0.046)	(0.038)
Gender (b.=male)		−0.217**	−0.038
		(0.075)	(0.062)
Age (Std.)		0.085*	0.105***
		(0.038)	(0.031)
Education (b.=low)		−0.461***	−0.337***
		(0.084)	(0.069)
Income (Deciles)		0.004	−0.022*
		(0.013)	(0.011)
Migration background (b.=no)		−0.126	−0.056
		(0.108)	(0.089)
Left-right (Std.)			1.408***
			(0.028)
In-group affect (Std.) X Rural residence	0.511***	0.494***	0.386***
	(0.078)	(0.084)	(0.069)
Out-group affect (Std.) X Rural residence	−0.751***	−0.721***	−0.500***
	(0.077)	(0.083)	(0.068)
Constant	5.801***	6.027***	6.040***
	(0.101)	(0.138)	(0.114)
Country fixed effects	Yes	Yes	Yes
Num.Obs.	6,430	5,474	5,474
R2	0.061	0.073	0.365
R2 Adj.	0.059	0.070	0.363

+ p < 0.1, * p < 0.05, ** p < 0.01, *** p < 0.001

Table A.28: Multilevel regression results: empty models of place-based affective polarisation and GAL-TAN voting.

	Place-based affective polarisation		GAL-TAN voting	
	NUTS-2	NUTS-3	NUTS-2	NUTS-3
	(1)	(2)	(3)	(4)
Constant	1.958***	1.831***	5.424***	5.410***
	(0.400)	(0.318)	(0.073)	(0.054)
σ_u^2	11.400	13.416	0.499	0.604
σ_e^2	497.129	494.686	7.505	7.441
ICC	0.022	0.026	0.062	0.075
N level-1: individuals	9,114	9,114	6,430	6,430
N level-2: regions	149	742	147	703

+ p < 0.1, * p < 0.05, ** p < 0.01, *** p < 0.001

Table A.29: Multilevel regression results: GAL-TAN voting on place-based affective polarisation, conditional on urban-rural self-classifications (random intercepts for NUTS-2 region).

	(1)	(2)	(3)	(4)	(5)	(6)	(7)
Thermometer differential (Std.)		–0.347***	–0.340***	–0.220***	–0.222***	–0.222***	–0.199***
		(0.044)	(0.047)	(0.039)	(0.039)	(0.039)	(0.041)
Rural residence (b.=urban residence)		0.248**	0.186+	0.136+	0.127	0.121	0.105
		(0.089)	(0.096)	(0.079)	(0.079)	(0.079)	(0.084)
Gender (b.=male)			–0.227**	–0.059	–0.055	–0.055	–0.060
			(0.074)	(0.062)	(0.062)	(0.062)	(0.065)
Age (Std.)			0.079*	0.099**	0.095**	0.095**	0.075*
			(0.038)	(0.031)	(0.031)	(0.031)	(0.033)
Education (b.=low)			–0.452***	–0.328***	–0.325***	–0.327***	–0.225**
			(0.083)	(0.069)	(0.069)	(0.069)	(0.072)
Income (Deciles)			0.008	–0.021+	–0.021+	–0.020+	–0.013
			(0.013)	(0.011)	(0.011)	(0.011)	(0.012)
Migration background (b.=no)			–0.104	–0.042	–0.042	–0.042	–0.057
			(0.108)	(0.089)	(0.089)	(0.089)	(0.095)
Left-right (Std.)				1.399***	1.396***	1.399***	1.394***
				(0.028)	(0.028)	(0.028)	(0.029)
Thermometer differential (Std.) X Rural residence		0.815***	0.784***	0.572***	0.572***	0.574***	0.507***
		(0.077)	(0.082)	(0.068)	(0.069)	(0.069)	(0.075)
Regional unemployment (Std.)					–0.098	–0.138+	–0.113
					(0.077)	(0.074)	(0.078)
Population Δ (Std.)						–0.175*	–0.296***
						(0.071)	(0.076)
EQI							0.253+
							(0.137)
Constant	6.089***	5.815***	6.018***	6.049***	5.961***	6.268***	6.540***
	(0.180)	(0.169)	(0.191)	(0.152)	(0.166)	(0.198)	(0.210)
Country fixed effects	Yes	Yes	Yes	Yes	Yes	Yes	Yes
σ_u^2	0.182	0.147	0.140	0.081	0.083	0.061	0.039
σ_e^2	7.511	7.357	7.233	4.968	4.958	4.965	4.834
ICC	0.024	0.020	0.019	0.016	0.016	0.012	0.008
N level-1: individuals	6,430	6,430	5,474	5,474	5,448	5,448	4,799
N level-2: regions	147	147	147	147	144	144	107

+ $p < 0.1$, * $p < 0.05$, ** $p < 0.01$, *** $p < 0.001$

References

Bornschier, S., Häusermann, S., Zollinger, D., and Colombo, C. (2021). How "Us" and "Them" Relates to Voting Behavior—Social Structure, Social Identities, and Electoral Choice. *Comparative Political Studies*, 54(12):2087–2122.

Charron, N., Lapuente, V., and Bauhr, M. (2024). The Geography of Quality of Government in Europe. Subnational Variations in the 2024 European Quality of Government Index and Comparisons With Previous Rounds. QoG Working Paper Series 2024:2, Department of Political Science, University of Gothenburg.

Hainmueller, J., Mummolo, J., and Xu, Y. (2019). How Much Should We Trust Estimates From Multiplicative Interaction Models? Simple Tools to Improve Empirical Practice. *Political Analysis*, 27(2):163–192.

Hooghe, L., Marks, G., Bakker, R., Jolly, S., Polk, J., Rovny, J., Steenbergen, M., and Vachudova, M. A. (2024). The Russian Threat and the Consolidation of the West: How Populism and EU-Skepticism Shape Party Support for Ukraine. *European Union Politics*, 25(3):459–482.

Munis, B. K. (2022). Us Over Here Versus Them Over There ... Literally: Measuring Place Resentment in American Politics. *Political Behavior*, 44(3):1057–1078.

Wagner, M. (2021). Affective Polarization in Multiparty Systems. *Electoral Studies*, 69(February):102199.
